# Protective Effect of Tetrahydroquinolines from the Edible Insect *Allomyrina dichotoma* on LPS-Induced Vascular Inflammatory Responses

**DOI:** 10.3390/ijms21103406

**Published:** 2020-05-12

**Authors:** InWha Park, Wonhwa Lee, Youngbum Yoo, Hyosoo Shin, Joonseok Oh, Hyelim Kim, Mi-Ae Kim, Jae Sam Hwang, Jong-Sup Bae, MinKyun Na

**Affiliations:** 1 College of Pharmacy, Chungnam National University, Daejeon 34134, Korea; inwha129@naver.com (I.P.); rimeeyo@gmail.com (H.K.); 2 Aging Research Center, Korea Research Institute of Bioscience and Biotechnology (KRIBB), Daejeon 34141, Korea; wonhwalee@kribb.re.kr (W.L.); ddq3416@gmail.com (Y.Y.); hysooo0914@gmail.com (H.S.); 3 Department of Chemistry, Yale University, New Haven, CT 06520, USA; joonseok.oh@yale.edu; 4 Department of Agricultural Biology, The National Academy of Agricultural Science, RDA, Wanju-gun 55365, Korea; kimma@korea.kr (M.-A.K.); hwangjs@korea.kr (J.S.H.); 5 College of Pharmacy, CMRI, Research Institute of Pharmaceutical Sciences, BK21 Plus KNU Multi-Omics Based Creative Drug Research Team, Kyungpook National University, Daegu 41566, Korea

**Keywords:** *Allomyrina dichotoma*, anti-inflammatory effect, dopamine derivative, NF-κB, tetrahydroquinoline

## Abstract

The larva of *Allomyrina dichotoma* (family Scarabaeidae) is an edible insect that is registered in the Korean Food Standards Codex as a food resource. The chemical study on the larvae of *A. dichotoma* resulted in the isolation of three new tetrahydroquinolines, allomyrinaines A–C (**1**–**3**), one new dopamine derivative, allomyrinamide A (**4**), and four known compounds (**5**–**8**). The structures were elucidated on the basis of 1D and 2D nuclear magnetic resonance (NMR) and MS spectroscopic data analysis. Allomyrinaines A–C (**1**–**3**) possessed three stereogenic centers at C-2, C-3, and C-4, whose relative configurations were determined by analyses of the coupling constants and the nuclear Overhauser enhancement spectroscopy (NOESY) data, as well as DP4+ calculation. The anti-inflammatory effects of compounds **1**–**4** were evaluated in human endothelial cells. Allomyrinaines A–C (**1**–**3**) could stabilize vascular barrier integrity on lipopolysaccharide (LPS)-induced vascular inflammation via inhibition of the nuclear factor-κB (NF-κB) pathway. The physiologically relevant concentration was confirmed by Q-TOF-MS-based quantitative analysis on allomyrinaines A–C in crude extract. This study suggests that allomyrinaines A–C (**1**–**3**) are bioactive constituents of *A. dichotoma* to treat vascular inflammatory disorder.

## 1. Introduction

Globally, the most commonly consumed insects are beetles (Coleoptera) [1]. *Allomyrina dichotoma* L. (family Scarabaeidae, order Coleoptera), a rhinoceros beetle, is mainly distributed in Korea, Japan, China, and Taiwan. Recently, the larva of *A. dichotoma* has been registered in the Korean Food Standards Codex as a new food resource. Researches have focused on this insect resource for the development of functional food and medicinal material. Chemical investigations of *A. dichotoma* reported that it contains general nutrients such as fatty acids and nucleosides [2,3]. A biological study of the larvae extract revealed hepatoprotective [4], anticancer [4], antidementia [5], antiobesity [6], and antioxidant [7] activities. Despite the potential application of *A. dichotoma* in the development of food and medicinal resource, there are few studies on the bioactive small molecules originating from this insect. The objective of this study was thus to explore the bioactive constituents from *A. dichotoma* larvae. 

The presence of lipopolysaccharide (LPS), a bacterial endotoxin, ranks the highest among the risk factors that contribute to lethal endotoxemia [8]. The integrity of vascular endothelium is essential for controlling the flux of proteins, fluids, and immune cells across vessels into tissues, and vascular endothelial dysfunction is a critical event in acute inflammation [9,10]. The systemic accumulation of LPS triggers leukocyte infiltration within the vascular wall and promotes vascular permeability [11]. Therefore, the maintenance of vascular integrity is crucial for vascular and tissue homeostasis.

Previous studies have shown that antidesmone (tetrahydroquinoline alkaloid) derived from *Antidesma membranaceum* (Euphorbiaceae) has an anti-inflammatory effect on LPS-induced RAW264.7 mouse macrophage cells [12]. The anti-inflammatory effects were regulated through the mitogen-activated protein kinase (MAPK)–nuclear factor-κB (NF-κB) signaling pathways. Herein, we report on the details of the isolation, structure elucidation of new tetrahydroquinolines (1–3), and a new dopamine derivative (4) as well as their vascular barrier protective effects on LPS-mediated vascular inflammatory responses in vitro and in vivo.

## 2. Results and Discussion

### 2.1. Identification of Isolated Compounds

**Figure 1:**
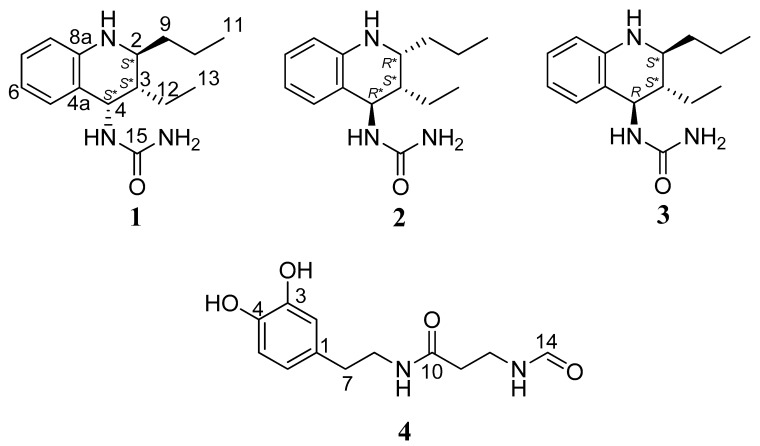
Chemical structures of tetrahydroquinolines (1–3) and a dopamine derivative (4).

Three new tetrahydroquinolines (1–3) and one new dopamine derivative (4) were isolated from *A. dichotoma* larvae, and their structures were established by spectroscopic techniques, especially detailed analyses of nuclear magnetic resonance (NMR) spectra (Figure 1). Compounds **1**–**3**, structurally based on diastereomeric tetrahydroquinolines, were obtained as colorless oil. Their molecular formulas were as C_15_H_23_N_3_O based on observed high resolution electrospray ionization mass (HRESIMS) ions (*m*/*z* 262.19 [M + H]^+^, 284.17 [M + Na]^+^) and their NMR information.

**Figure 2:**
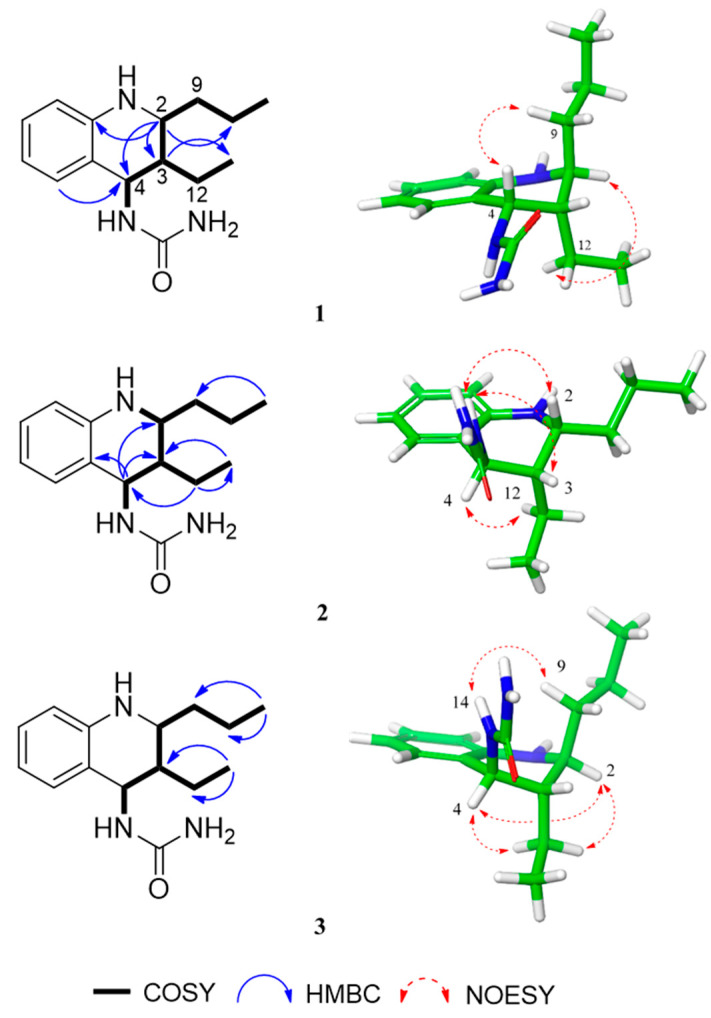
The ^1^H-^1^H correlation spectroscopy (COSY), heteronuclear multiple bond correlation (HMBC), and selected key nuclear Overhauser enhancement spectroscopy (NOESY) correlations of 1–3.

The ^1^H NMR spectrum of **1** measured in methanol-*d*_4_ showed the diagnostic signals of one *ortho*-disubstituted benzene (*δ*_H_ 7.08, 1H, d, *J* = 7.47 Hz, H-5; *δ*_H_ 6.92, 1H, m, H-7; *δ*_H_ 6.50, 2H, overlap, H-8 and H-6), three methine (*δ*_H_ 4.99, 1H, d, *J* = 4.24 Hz, H-4; *δ*_H_ 3.14, 1H, dd, *J* = 6.54, 11.87 Hz, H-2; *δ*_H_ 1.63, 1H, qd, *J* = 4.24, 6.54 Hz, H-3), and three methylene (*δ*_H_ 1.55, 1H, m, H_2_-9; *δ*_H_ 1.46, 1H, m, H_2_-10; *δ*_H_ 1.32, 2H, m, H_2_-12) (Table 1). The proton signals at *δ*_H_ 1.00 (3H, t, *J* = 6.61 Hz, H_3_-13) and 0.97 (3H, t, *J* = 6.40 Hz, H_3_-11) indicated the presence of two terminal methyl groups. A urea and two nitrogen-linked methine carbons were observed at *δ*_C_ 161.8, 52.5, and 47.8. Analysis of the ^1^H-^1^H correlation spectroscopy (COSY) data for **1** revealed two individual spin systems: one propyl (H-2–H_2_-9–H_2_-10–H_3_-11) group and one ethyl (H-3–H_2_-12–H_3_-13) group (Figure 2). Urea, propyl, and ethyl moieties were connected to C-4, C-3, and C-2, respectively, based on the heteronuclear multiple bond correlation (HMBC) cross-peaks (Figure 2). According to the NMR and MS spectroscopic data, the planar structure was defined to be a *N*-(1,2,3,4-tetrahydro-2-propyl-3-ethyl-4-quinolinyl)-urea. Compound 1 possessed three stereogenic centers at C-2, C-3, and C-4 comprising the piperidine core. The relative configureuration of the piperidine ring in 1 could be established from ^1^H–^1^H coupling constants and nuclear Overhauser enhancement spectroscopy (NOESY) data analysis (Figure 2). The small coupling constants between H-4 (*J* = 4.24 Hz) and H-3 indicated a gauche conformation with a torsion angle of approximately 60°. Having established the orientations of H-3 and H-4, there are four possible configurations that are feasible for C-2 to C-4, i.e., axial–equatorial–axial, equatorial–equatorial–axial, axial–equatorial–equatorial, and equatorial–equatorial–equatorial. In the NOESY spectrum, the nuclear Overhauser effect (NOE) correlations, H-4/H_2_-9 and H-2/H_2_-12, indicated that H-2, H-3, and H-4 were equatorial–equatorial–axial orientations (Figure 2). To clarify the structure, aprotic solvent was employed. A set of additional NOE correlations between NH-14 and H_2_-12 are present in the acetone-*d*_6_ (Table 2) Thus, compound 1 was assigned as *N*-[(2S*,3S*,4S*)-1,2,3,4-tetrahydro-2-propyl-3-ethyl-4-quinolinyl]-urea, namely, allomyrinaine A.

The ^1^H NMR spectroscopic data of 2 were similar to those of 1. The significant difference was the ^13^C chemical shift of the C-4 stereogenic center (*δ*_C_ 50.7) deshielded 2.9 ppm compared to that of 1 (*δ*_C_ 47.8), indicating that 2 would be a diastereomer of 1. The *J*_H-3/H-4_ value of 3.55 Hz also shows that these two protons are a gauche conformation. NOESY correlations are observed for NH-14/H-2 and H-4/H_2_-12, implying that H-3/H-4, and H-2/H-4 were oriented as shown in Figure 2. Finally, the structure of 2 was determined as *N*-[(2R*,3S*,4R*)-1,2,3,4-tetrahydro-2-propyl-3-ethyl-4-quinolinyl]-urea, and it was named allomyrinaine B. 

The ^1^H and ^13^C NMR spectroscopic data of **3** were similar to those of **2**, except for the deshielded signal at *δ*_C_ 54.2. The NOE correlations between H-4 and H-2 proved that these protons were cofacial (axial–axial or equatorial–equatorial). Additionally, the NOE correlations of H-2 and H-4 to H_2_-12 suggested that the cyclohexane ring occupied either 2S*,3S*,4R* (3a) or 2S*,3R*,4R* (3b) (Figure S31). The relative configuration at C-3 was conducted by applying computational NMR chemical shift calculations supported by DP4+ analysis [13]. The ^1^H and ^13^C NMR chemical shift values of the diastereomers 3a and 3b were compared with those of the experimental NMR data of 3 using the improved probability DP4+ method. The statistical analyses revealed that the probability of diastereomer 3a (2S*,3S*,4R*) is 100.0% in the consideration of both ^1^H and ^13^C NMR chemical shift values. Collectively, the structure of 3 was established as *N*-[(2S*,3S*,4R*)-1,2,3,4-tetrahydro-2-propyl-3-ethyl-4-quinolinyl]-urea and assigned the preliminary name of allomyrinaine C. Their electronic circular dichroism (ECD) data did not exhibit significant Cotton effects and specific rotation values were small, indicating that they were presumably scalemic mixtures. 

Compound 4 was obtained as brown oil. The molecular formula was determined to be C_12_H_16_N_2_O_4_ by HRESIMS (*m*/*z* 275.1003 [M + Na]^+^, calcd 275.1008) combined with the ^1^H and ^13^C NMR spectra. ^1^H and HSQC data of 4 disclosed the presence of two OH groups (*δ*_H_ 8.74, 1H, brs, 3-OH; 8.65, 1H, brs, 4-OH), one trisubstituted benzene (*δ*_H_ 6.62, 1H, d, *J* = 7.96 Hz, H-5; *δ*_H_ 6.56, 1H, d, *J* = 1.93 Hz, H-2; *δ*_H_ 6.42, 1H, dd, *J* = 7.96, 1.93 Hz, H-6), and four methylene groups (*δ*_H_ 3.26, q, *J* = 6.86 Hz, H-12; *δ*_H_ 3.17, q, *J* = 6.09 Hz, H-8; *δ*_H_ 2.51, m, H-7; *δ*_H_ 2.23, t, *J* = 6.86 Hz, H-11). The ^13^C NMR spectrum displayed 12 carbon signals allocated to a nitrogenated tertiary sp^2^ carbon (*δ*_C_ 170.0), a nitrogenated terminal aldehyde carbon (*δ*_C_ 161.1), six benzene carbons (*δ*_C_ 145.0, 143.5, 130.2, 119.2, 115.9, 115.5), and four methylene carbons (*δ*_C_ 40.6, 35.2, 34.6, 33.9). The ^1^H and ^13^C NMR spectra were similar to those of *N*-acetyldopamine, except for the *N*-ethylformamide group [14]. Key HMBC correlations from H-7 to C-2/C6, from H-8/H-11 to C-10, and from H-12 to C-14 supported the presence of a formylamino-*N*-prophyl-propanamide moiety, as well as its connection to C-1. Thus, the structure of 4 was defined as *N*-(3,4-dihydroxyphenethyl)-1-formamidopropanamide, and it was named allomyrinamide A. The four known analogues were identified as arbutin (5) [15], cyclo(L-Val-L-Pro) (6) [16], inosine (7) [2], and 1,2-benzenediol (**8**) [17], respectively, by extensive spectroscopic analysis and by comparing their NMR data with those reported in the literature. 

### 2.2. Anti-Inflammatory Activity

#### 2.2.1. Effect of New Compounds (1–4) on Lipopolysaccharide (LPS)-mediated Vascular Inflammatory Responses

Vascular inflammation plays an important role in the pathogenesis of multiple organ damage due to septic shock. Gram-negative LPS is one of the major inflammatory pathogens of sepsis [18]. LPS directly activates the vascular endothelium and monocyte/macrophage to induce inflammation [19]. Endothelial cells elicit a series of specific cellular responses, including increased cell adhesion molecule and inflammatory cytokine/chemokine expression [20]. This leads to vascular disruption and the recruitment of leukocytes to enhance the excess inflammatory responses [19]. 

**Figure 3:**
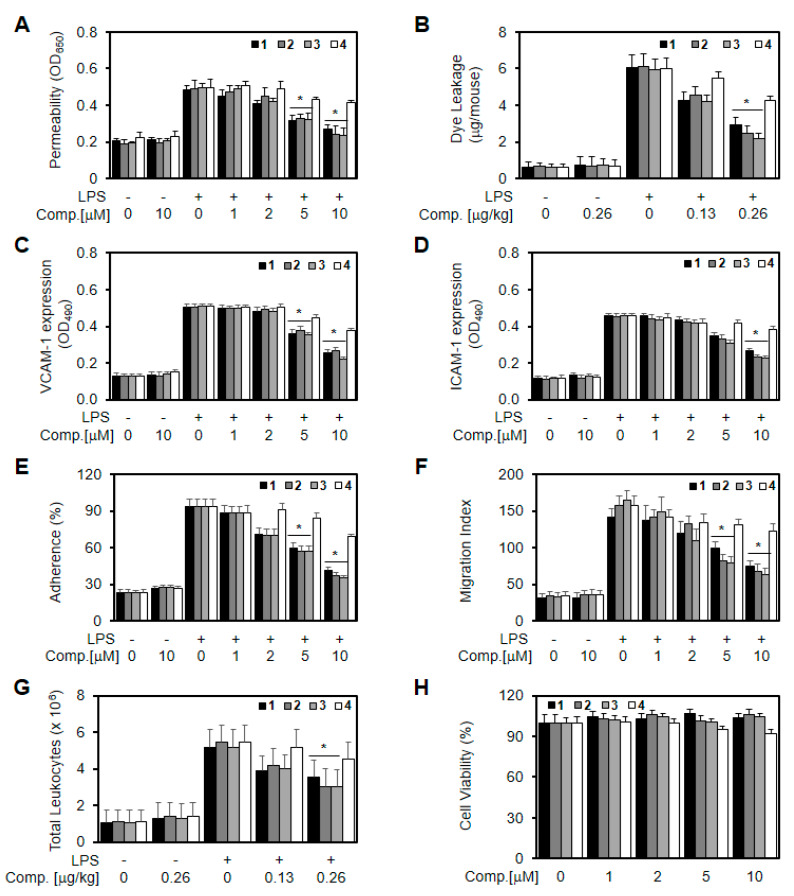
Effects of compounds (**1**–**4**) on lipopolysaccharide (LPS)-mediated vascular inflammatory responses. (**A**) Effect of various concentrations of compounds (1–4) on LPS-induced (100 ng/mL, 4 h) barrier disruption was monitored by the flux of Evans blue bound albumin across human umbilical vein endothelial cells (HUVECs); (**B**) Effect of compounds (1–4) on LPS (15 mg/kg, i.p.)-induced vascular permeability in mice was examined by the flux of Evans blue in mice (expressed μg/mouse, *n* = 5); LPS-mediated (100 ng/mL) expression of vascular cell adhesion molecule-1 (VCAM-1) (**C**) and intercellular adhesion molecule-1 (ICAM-1) (**D**) in HUVECs was analyzed after treating monolayers with each compound (10 μM each) by whole cell enzyme-linked immunosorbent assay (ELISA); (**E**) LPS-mediated (100 ng/mL) adherence of monocytes to HUVEC monolayers was analyzed after treating cells with each compound; (**F**) LPS-mediated (100 ng/mL) migration of human neutrophils through HUVEC monolayers was analyzed after treating cells with each compound; (**G**) The effects of each compound on LPS (15 mg/kg, i.p.)-induced leukocyte migration in mice (expressed ×10^6^, *n* = 5); (**H**) The effects of each compound on cell viability were evaluated using CCK8 assays. The results are expressed as the means ± SEM of three independent experiments. * *p* < 0.05 vs. LPS.

A permeability assay was used to determine the effects of each compound on the barrier integrity of human umbilical vein endothelial cells (HUVECs). Treatment with each compound (10 µM) alone did not result in an alteration of barrier integrity (Figure 3a). On the other hand, LPS is known to cleave and disrupt endothelial barrier integrity [21]. Thus, HUVECs were treated with various concentrations of each compound for 6 h after the addition of LPS (100 ng/mL) for 4 h. The results shown in Figure 3a indicate that new tetrahydroquinolines (1–3) inhibit the LPS-mediated hyperpermeability in endothelial cells, with the optimal effect occurring at a concentration above 5 µM. To confirm this effect in vivo, the LPS-induced vascular permeability in mice was assessed. As shown in Figure 3B, the new tetrahydroquinolines (1–3) induced a marked inhibition of the peritoneal leakage of dye induced by LPS. Assuming that the average weight of a mouse is 20 g and the average blood volume is 2 mL, then the amount of each compound injected (0.13, 0.26 mg/kg) was equivalent to 5 or 10 µM in peripheral blood. 

Cell adhesion molecules (CAMs) such as vascular cell adhesion molecule-1 (VCAM-1) and intercellular adhesion molecule-1 (ICAM-1) play a pivotal role in the process of vascular inflammatory processes [21]. The adhesion of circulating leukocytes to the vascular endothelium is a fundamental step in leukocyte extravasation during inflammation, and CAMs mediate this process [21]. Therefore, inhibiting the expression of CAMs in vascular endothelial cells is considered to be a promising therapeutic approach for treating vascular inflammatory diseases. We found that LPS induced the upregulation of the surface protein expressions of VCAM-1 and ICAM-1 (Figure 3C,D) and that each compound inhibited this effect, suggesting that the inhibitory effects of each compound on the expression of CAMs are mediated via the attenuation of the LPS signaling pathway by each compound. Experiments on CAMs are widely used in vitro for studying the regulation of the interactions between leukocytes and endothelial cells [22]. In addition, an elevated expression of CAMs was found to correspond well with the enhanced binding of leukocytes cells to LPS-activated endothelial cells, followed by their migration. Moreover, treatment with compounds 1–3 resulted in the downregulation of neutrophils adherence and their subsequent migration across activated endothelial cells in a concentration-dependent manner (Figure 3e,f). These results suggest that compounds 1–3 downregulate the pro-inflammatory signaling effect of released LPS, thereby inhibiting the amplification of inflammatory pathways by nuclear cytokines. To confirm this effect in vivo, we examined LPS-induced leukocyte migration in mice. LPS was found to stimulate leukocyte migration into the peritoneal cavities of mice, and treatment with each compound resulted in a significant reduction of peritoneal leukocyte counts (Figure 3g). Therefore, in the current study, treatment with compounds 1–3 resulted in the downregulation of LPS-induced levels of VCAM-1 and ICAM-1, suggesting that compounds 1–3 inhibit the adhesion and migration of leukocytes to an inflamed endothelium. To test the cytotoxicity of each compound, cellular viability assays were performed in HUVECs treated with each compound for 48 h. At concentrations up to 10 µM, each compound did not affect cell viability (Figure 3h). Therefore, the results obtained in this study suggest that the new tetrahydroquinolines (1–3) have potential as therapeutic agents against vascular inflammatory diseases. 

#### 2.2.2. Effect of New Compounds (1–3) on the LPS-stimulated Activation of Nuclear Factor-κB (NF-κB), Production of Interleukin-1β (IL-1β)/Tumor Necrosis Factor-α (TNF-α), and Phosphorylation of p38 MAPK

**Figure 4:**
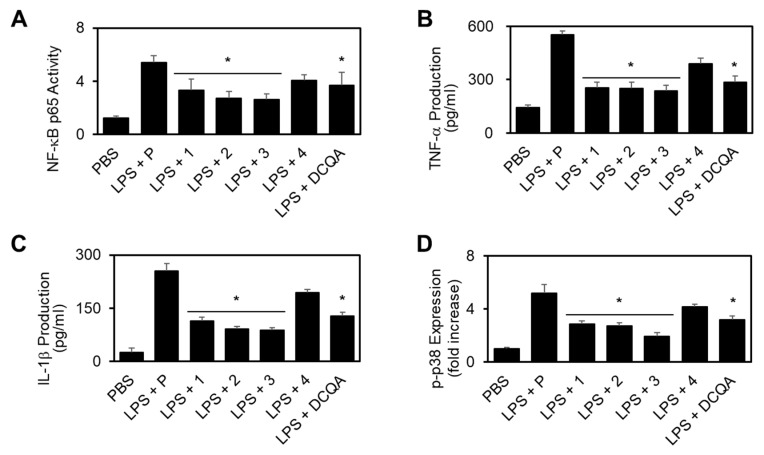
Effect of new compounds (**1**–**4**) on the LPS-stimulated activation of nuclear factor-κB (NF-κB), the production of tumor necrosis factor-α (TNF-α)/ interleukin-1β (IL-1β), and the phosphorylation of p38 mitogen-activated protein kinase (MAPK). First, 20 μM of 3-5-di-*O*-dihydrocaffeoylquinic acid (DCQA) was used as positive control. (**A**) LPS (100 ng/mL)-mediated activation of NF-κB p65 in HUVECs was analyzed after the treatment of cells with 10 μM of each compound for 6 h; LPS (100 ng/mL)-mediated production of TNF-α (**B**) or IL-6 (**C**) in HUVECs was analyzed after the treatment of cells with the indicated concentrations of each compound for 6 h; (**D**) HUVECs were activated with LPS (100 ng/mL), followed by treatment with each compound at different concentrations for 6 h. The effects of each compound on the LPS-mediated expression of phospho-p38 were determined by ELISA. * *p* < 0.05 vs. LPS.

NF-κB activation is required for pro-inflammatory responses, and the **3** most important providers of inflammatory signals in endothelial cells are nuclear factor-κB (NF-κB), tumor necrosis factor-α (TNF-α), and interleukin-1β (IL-1β) [21]. Therefore, we tested the effects of each compound on the activation of NF-κB by LPS. As shown in Figure 4a, the activation of NF-κB was increased by LPS, and these increases were significantly reduced by each compound. Therefore, these results indicate that compounds **1**–**3** can regulate important signals that induce pro-inflammatory responses in human endothelial cells. As a positive control, 3-5-di-*O*-dihydrocaffeoylquinic acid (DCQA), a compound previously identified to be effective in vascular inflammation, was used [23]. The suppression of vascular inflammation inducing NF-κB, TNF-α, IL-1β, and p-p38 expression with 20 µM of compound was confirmed through previous studies [23]. 

Based on these results, we hypothesized that each compound might inhibit the expression or activity of these pro-inflammatory molecules. To investigate the potential effects of compounds **1**–**3** on the production of the pro-inflammatory cytokines, TNF-α and IL-1β, HUVECs were incubated with each compound for 6 h after LPS activation, and then TNF-α and IL-1β levels in the culture media were measured via enzyme-linked immunosorbent assay (ELISA). Levels of TNF-α and IL-1β showed an increase in LPS-stimulated endothelial cells; these increases were significantly reduced by compounds 1–3 (Figure 4b,c), indicating that compounds 1–3 can regulate the most important signals that induce pro-inflammatory responses in human endothelial cells. 

LPS is known to induce pro-inflammatory responses by promoting the phosphorylation of p38 mitogen-activated protein kinase (MAPK) [24]. To determine whether each compound inhibits p38 MAPK in LPS-activated HUVECs, HUVECs were pre-incubated with each compound and then activated with LPS, followed by the examination of phosphor p38 MAPK levels by ELISA. As shown in Figure 4D, LPS induced an upregulated expression of phosphorylated p38, and the significant inhibition of this upregulation was observed by treatment with compounds **1**–**3**.

#### 2.2.3. Protective Effect of Each Compound in Cecal Ligation and Puncture (CLP)-Induced Septic Mice

**Figure 5:**
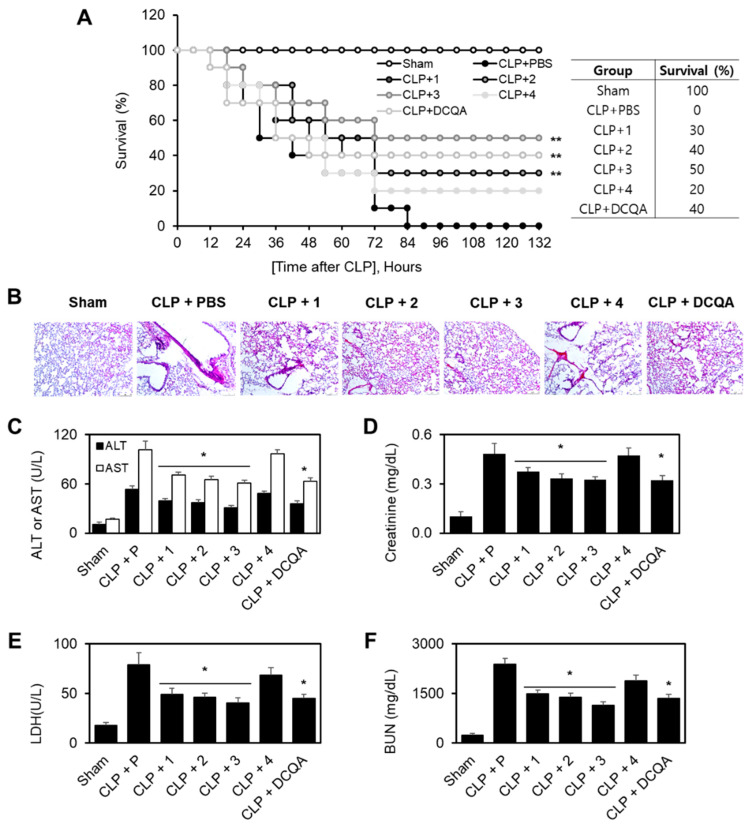
Effects of each compound on cecal ligation and puncture (CLP)-induced mortality and pulmonary injury. (**A**) Male C57BL/6 mice (*n* = 10) were intravenously treated with each compound (0.26 mg/kg) or DCQA (1 mg/kg, positive control at 12 and 50 h after CLP. Animal survival was monitored every 6 h after CLP for 132 h. Kaplan–Meier survival analysis was performed for evaluating of the overall survival rates; (**B**) Male C57BL/6 mice underwent CLP and were administered each compound (0.26 mg/kg) intravenously at 12 and 50 h after CLP (*n* = 5). Mice were killed 96 h after CLP. Photomicrographs of lung tissues (H&E staining, 200×, White scale bar = 50 µm). The illustrations show representative images from three independent experiments; (**C**–**F**) The same as (**B**,**C**) except that mice were bled to death. Aspartate transaminase (AST) (**C**), alanine transaminase (ALT) (**C**), creatinine (**D**), blood urea nitrogen (BUN) (**E**), and lactate dehydrogenase (LDH) (**F**) levels in the plasma were measured. The results are expressed as the means ± SD of five independent experiments (*n* = 5). * *p* < 0.05 vs. the CLP group.

Sepsis is a systemic response to serious infections, and it has a poor prognosis when it is associated with organ dysfunction, hypoperfusion, or hypotension [22]. Based on our previous findings, it was hypothesized that each compound may reduce the mortality in a cecal ligation and puncture (CLP)-induced sepsis mouse model. To test this hypothesis, each compound was administered to mice after a CLP procedure. Twenty-four h after the operation, the mice showed signs of sepsis, such as shivering, bristled hair, and weakness. The administration of each compound (0.26 mg/kg) 12 h after CLP did not prevent CLP-induced death, so each compound (0.26 mg/kg) was administered twice (12 and 50 h after CLP). Administration of two doses of compounds **1**–**3** resulted in an increase in the survival rate from 40% to 60%, based on Kaplan–Meier survival analysis (*p* < 0.0001; Figure 5a). This marked survival benefit that was achieved after the administration of two doses of compounds **1**–**3** suggested that the protection of LPS-mediated vascular inflammatory responses could be a therapeutic strategy for the management of sepsis and septic shock. We compared DCQA with improved survival in CLP-induced septic mice as a positive control [23].

To confirm the protective effects of compounds **1**–**3** against CLP-induced death, its effects were examined upon CLP-induced pulmonary injury. In the sham treatment group, there were no significant differences between the lungs of the treated and untreated mice under the light microscope. In the untreated CLP group, interstitial edema with massive infiltration of inflammatory cells into the interstitium and alveolar spaces was observed, and the pulmonary architecture was severely damaged (Figure 5B). Systemic inflammation during sepsis commonly causes multiple organ failure, and the liver and kidneys are the major target organs [25]. CLP resulted in a significant increase in the plasma levels of alanine transaminase (ALT) and aspartate transaminase (AST) (markers of hepatic injury; Figure 5C), as well as creatinine and blood urea nitrogen (BUN) (markers of renal injury; Figure 5D,E). Compounds 1–3 were able to reverse all of these abnormalities. The levels of another important marker of tissue injury, lactate dehydrogenase (LDH), were also reduced by compounds **1**–**3** in CLP-operated mice (Figure 5F).

#### 2.2.4. Quantitative Analysis of 1–3

The physiologically relevant concentration was confirmed by quadruple time-of-flight mass (Q-TOF-MS)-based quantitative analysis on allomyrinaines A–C (1–3) in crude extract. Based on the calibration curves, the concentrations of allomyrinaines A, B, and C in the extract were estimated to be 12, 15, and 16 μM, respectively (Figure S44). The micromolar range contents of allomyrinaines A–C in the extract represent the biological activity of the edible insect *A. dichotoma*. 

## 3. Materials and Methods

### 3.1. General Experimental Procedures

Optical rotations were measured on a JASCO DIP-370 (Tokyo, Japan) automatic digital polarimeter. UV spectra were recorded on a Shimadazu SPD-M20A PDA detector, and IR spectra were obtained on a Bruker ALPHA FT-IR spectrometer. Nuclear magnetic resonance (NMR) experiments were conducted using Bruker DMX 300 (^1^H-300 MHz, ^13^C-75 MHz) and Bruker DMX 600 (^1^H-600 MHz, ^13^C-150 MHz) spectrometers. Mass spectral data were obtained on a SYNAPT G2 Waters mass spectrometer (Manchester, UK). Thin layer chromatography (TLC) was executed on glass plates precoated with silica gel 60 F_254_ and RP-18 F_254_ (20 × 20 cm, 200 μm, 60 Å, Merck, Darmstadt, Germany). Vacuum liquid chromatography (VLC) was implemented on silica gel (70–230 mesh, Merck), and medium-pressure liquid chromatography (MPLC) was performed utilizing a Biotage Isolera^TM^ apparatus equipped with a reversed phase C_18_ SNAP Cartridge KP-C18-HS (120 g and 400 g, Biotage AB, Uppsala, Sweden). Preparative high-performance liquid chromatography (HPLC) was performed using a Gilson system with a UV detector and a Phenomenex Kinetex C_18_ column (250 × 21.2 mm, 5 μm), a Phenomenex Kinetex Biphenyl column (250 × 21.2 mm, 5 μm), or a Hector column (250 × 21.2 mm, 5 μm). Concentrations of compounds 1–3 in the crude extract were deduced using Q-TOF-MS analysis (Agilent iFunnel 6550 quadrupole time of-flight MS instrument fitted with an electrospray ionization (ESI) source coupled to an Agilent 1290 Infinity HPLC system). Protonated ions of title compounds were extracted in the crude sample, and their areas under curves were plotted with respect to calibration curves with different concentrations of pure compounds 1–3 (Figure S44).

### 3.2. Insect Material

The larvae of *A. dichotoma* were collected from the National Academy of Agricultural Science, RDA, Korea and were identified by M-A. Kim (National Academy of Agricultural Science). A voucher specimen (CNU-INS201603) was deposited at the Laboratory of Pharmacognosy of the College of Pharmacy, Chungnam National University, Daejeon, Korea.

### 3.3. Extraction and Isolation

The dried larvae of the *A. dichotoma* (4.5 kg) were refluxed with 1% acetic acid in EtOH (35 L) for 16 h. The concentrated extract (672.0 g) was subjected to silica gel vacuum liquid chromatography (VLC) and was eluted with *n*-hexane/EtOAc (10:0, 8:2, 6:4, 2:8) and CH_2_Cl_2_/MeOH (10:0, 8:2, 6:4, 2:8, 0:10) to yield 9 fractions (AD1 to AD9). AD7 (16.0 g) was fractionated by VLC over silica gel using a stepwise gradient elution (*n*-hexane/EtOAc/MeOH) to obtain 6 fractions (AD7-1 to AD7-6). Compound 8 (38.0 mg) was separated from fraction AD7-3 (1.3 g) using medium-pressure liquid chromatography (MPLC) with a gradient solvent system MeOH/H_2_O (1:9 → 8:2). AD7-4 (5.7 g) was subjected to C_18_ MPLC using a gradient of MeOH/H_2_O (1:9 → 6:4; 5.5 L, 6:4 → 10:0; 1 L) to obtain 8 subfractions (AD7-4-1 to AD7-4-8). AD7-4-5 (288.7 mg) was purified by employing preparative HPLC with a gradient elution of MeOH/H_2_O (1:9 → 2:8; 10 min, 2:8 → 2:8; 20 min, 2:8 → 3:7; 25 min, 3:7 → 3:7; 60 min) to produce 6 (43.1 mg, *t*_R_ = 38 min). Among the remaining fractions, AD9 (103.7 g) exhibited aromatic resonances (*δ*_H_ 6.5-8.0) in its ^1^H NMR spectrum. Therefore, AD9 was subjected to VLC on silica gel by gradient elution using EtOAc/MeOH (10:0, 8:2, 6:4, 4:6, 2:8, 0:10) as solvents to acquire 6 fractions (AD9-1 to AD9-6). AD9-3 (12.0 g) was divided into 8 subfractions (AD9-3-1 to AD9-3-8) by using C_18_ MPLC eluted with a gradient of MeOH/H_2_O (10:90 → 100:0). AD9-3-2 (494.0 mg) was further separated by using HPLC (MeOH/H_2_O, 10:90) to produce 5 (36.1 mg, *t*_R_ = 19.8 min). Compound 7 (26.4 mg, *t*_R_ = 36.2 min) was isolated from AD9-3-3 (584.2 mg) by employing HPLC elution with MeOH/H_2_O (5:95 → 10:90). AD9-3-4 (1.0 g) was separated by biphenyl column HPLC with MeOH/H_2_O (10:90 → 30:70, 20 min; 30:90 → 30:90, 60 min) gradient eluted to give 11 subfractions (AD9-3-4-1 to AD9-3-4-11). Compound 4 (6.2 mg, *t*_R_ = 38.9 min) was purified from AD9-3-4-8 (40.1 mg) by utilizing HPLC elution with an isocratic condition (MeOH/H_2_O, 2:8). AD9-3-6 (159.4 mg) was purified by reversed-phase preparative HPLC with a gradient elution of MeCN/H_2_O (2:8 → 4:6; 30 min, 4:6 → 5:5; 60 min, 9:1 → 9:1; 75 min) to produce 3 (2.0 mg, *t*_R_ = 62.0 min), 2 (4.6 mg, *t*_R_ = 64.5 min), and 1 (3.4 mg, *t*_R_ = 68.2 min).

#### 3.3.1. Allomyrinaine A (1)

Colorless oil; ${\left[\alpha \right]}_{D}^{19}$ −44.0 (*c* 0.05, MeOH); UV (MeOH) *λ*_max_ (log *ε*) 250 (2.94) nm; FT-IR (ATR) ν_max_ 3320, 2942, 2831, 1449, 1115, 1021 cm^–1^; ^1^H and ^13^C NMR data, see Table 1 and Table 2; HRESIMS *m*/*z* 284.1737 [M + Na]^+^, (calcd for C_15_H_23_N_3_ONa, 284.1739). 

#### 3.3.2. Allomyrinaine B (2)

Colorless oil; ${\left[\alpha \right]}_{D}^{18}$ 14.4 (*c* 0.05, MeOH); UV (MeOH) *λ*_max_ (log *ε*) 246 (2.64) nm; FT-IR (ATR) ν_max_ 3320, 2942, 2831, 1449, 1114, 1021 cm^–1^; ^1^H and ^13^C NMR data, see Table 1 and Table 2; HRESIMS *m*/*z* 262.1919 [M + H]^+^, 284.1740 [M + Na]^+^, (calcd for C_15_H_24_N_3_O, 262.1919, C_15_H_23_N_3_ONa, 284.1739).

#### 3.3.3. Allomyrinaine C (3)

Colorless oil; ${\left[\alpha \right]}_{D}^{18}$ −16.5 (*c* 0.04, MeOH); UV (MeOH) *λ*_max_ (log *ε*) 247 (2.94) nm; FT-IR (ATR) ν_max_ 3321, 2942, 2831, 1450, 1114, 1020 cm^–1^; ^1^H and ^13^C NMR data, see Table 1 and Table 2; HRESIMS *m*/*z* 262.1915 [M + H]^+^, 284.1736 [M + Na]^+^, (calcd for C_15_H_24_N_3_O, 262.1919, C_15_H_23_N_3_ONa, 284.1739).

#### 3.3.4. Allomyrinamide A (4)

Brown oil; UV (MeOH) *λ*_max_ (log *ε*) 280 (3.62) nm; FT-IR (ATR) ν_max_ 3316, 2942, 2831, 1660, 1448, 1415, 1115, 1020 cm^–1^; ^1^H NMR (600 MHz, DMSO-*d*_6_) *δ*_H_ 8.74 (1H, brs, 3-OH), 8.65 (1H, brs, 4-OH), 7.96 (1H, s, H-14), 7.90 (1H, m, NH-9), 6.62 (1H, d, *J* = 7.96 Hz, H-5), 6.56 (1H, d, *J* = 1.93 Hz, H-2), 6.42 (1H, dd, *J* = 1.93, 7.96 Hz, H-6), 3.26 (2H, q, *J* = 6.86 Hz, H-12), 3.17 (2H, q, *J* = 6.09 Hz, H-8), 2.51 (1H, m, H-7, overlapped), 2.23 (2H, t, *J* = 6.86 Hz, H-11); ^13^C NMR (150 MHz, DMSO-*d*_6_) *δ*c 170.0 (C-10), 161.1 (C-14), 145.0 (C-3), 143.5 (C-4), 130.2 (C-1), 119.2 (C-6), 115.9 (C-2), 115.5 (C-5), 40.6 (C-8), 35.2 (C-11), 34.6 (C-7), 33.9 (C-12); HRESIMS *m*/*z* 253.1185 [M + H]^+^, 275.1003 [M + Na]^+^ (calcd for C_12_H_17_N_2_O_4_, 253.1188, C_12_H_16_N_2_O_4_Na, 275.1008).

### 3.4. Computational NMR Chemical Shift Calculations

Conformational searches were carried out using the MacroModel (Version 9.9, Schrödinger LLC, New York, NY, USA) program interfaced in Maestro (Version 11.8, Schrödinger LLC) with a mixed torsional/low-mode sampling method. Advanced conformational searches were performed in the MMFF force field, in the gas phase with a 50 kJ/mol energy window and 10,000 maximum iterations based on the original authors’ recommendations [13]. NMR chemical shift calculations of all conformers within 10 kJ/mol of the relative energy were implemented in the Gaussian 09 package (Gaussian Inc., Wallingford, CT, USA) without geometry optimization in the B3LYP functional at the 6-31G(d,p) level. Chemical shift values were determined according to the equations. The calculated NMR properties were averaged according to the Boltzmann populations, and the conformers with more than 1% population were used for calculations of DP4+ probability analysis facilitated by the Excel sheet provided by Grimblat et al. [13,26].

### 3.5. Biological Activities

Detailed methods for the biological evaluation in this study are provided in Supplementary data.

## 4. Conclusions

The larvae of *A. dichotoma* have attracted attention in the development of food and medicinal materials because of various biological activities that include hepatoprotective [4], anticancer [4], antidementia [5], antiobesity [6], and antioxidant [7] activities. However, there are few chemical studies or bioactive constituents on this insect material. In this study, we have isolated three new tetrahydroquinolines (allomyrinaines A–C), one dopamine derivative (allomyrinamide A), and four known compounds [arbutin, cyclo(L-Val-L-Pro), inosine, 1,2-benzenediol] from the larvae of *A. dichotoma*. Their structures were elucidated by analyses of spectroscopic data, including 1D and 2D NMR and MS spectra. The stereogenic centers of allomyrinaines A–C (1–3) at C-2, C-3, and C-4 were determined by analyses of the coupling constants and the NOESY data, as well as DP4+ calculation. Recently, tetrahydroquinoline have been reported to have anti-inflammatory activity modulating inflammatory mediators [12,27]. We have investigated the anti-inflammatory effects of new compounds 1–4 in LPS-mediated human endothelial cells. Allomyrinaines A–C (1–3) inhibited LPS-mediated barrier disruption by increasing barrier integrity and inhibiting the expression of CAMs; compounds 1–3 also reduced neutrophils adhesion and migration toward HUVECs. These barrier protective effects of allomyrinaines A–C (1–3) were confirmed in a mouse model, in which treatment with compounds 1–3 resulted in the reduction of CLP-induced mortality and lung damage. This is the first report on the discovery of new tetrahydroquinolines (allomyrinaines A–C) and a dopamine derivative from *A. dichotoma* larvae. The biological effects of allomyrinaines A–C (1–3) on LPS-induced vascular inflammatory responses have been demonstrated, in which compounds 1–3 inhibit adhesion and the migration of leukocytes to an inflamed endothelium. In association with the protective effects of allomyrinaines A–C (1–3) on LPS-mediated vascular barrier disruption, intravenous administration of compounds 1–3 resulted in an increase in the survival rate from 40% to 60% (*p* < 0.0001; Figure 5A). Our findings suggest that allomyrinaines A–C are the bioactive constituents of *A. dichotoma* that are capable of modulating vascular inflammatory diseases, which may warrant the development of this edible insect as a functional food/supplementary resource.

## Figures and Tables

**Table 1:** ^1^H and ^13^C NMR spectroscopic data for compounds **1**–**3** (600 MHz, methanol-*d*_4_).

Position	1		2		3	
	*δ*_H_, mult (*J* in Hz)	*δ*_C_	*δ*_H_, mult (*J* in Hz)	*δ*_C_	*δ*_H_, mult (*J* in Hz)	*δ*_C_
2	3.14, dd (6.54, 11.87)	52.5	3.28, m	51.1	3.21, td (4.14, 7.43)	54.2
3	1.63, qd (4.24, 6.54)	42.7	1.68, m	44.2	1.67, qd (5.46, 7.43)	44.3
4	4.99, d, (4.24)	47.8	4.66, d (3.55)	50.7	4.70, d (5.46)	50.8
4a	-	122.5	-	121.2	-	123.3
5	7.08, d (7.47)	128.6	7.08, dd (1.02, 7.82)	131.7	7.04, d (7.51)	129.5
6	6.50, m, overlap	117.0	6.56, m, overlap	117.8	6.56, m, overlap	117.8
7	6.92, m	128.9	6.96, m	129.1	6.93, m	128.8
8	6.50, m, overlap	114.7	6.56, m, overlap	115.6	6.56, m, overlap	115.4
8a	-	145.5	-	146.3	-	145.9
9	1.55, m	38.3	1.53, m	34.8	1.62/1.56, m	37.4
10	1.46, m	19.4	1.49/1.40, m	20.4	1.52, m	23.4
11	0.97, t (6.40)	14.6	0.99, m, overlap	14.5	0.97, t (7.55), overlap	14.6
12	1.32, m	20.7	1.44/0.99, m	19.4	1.50/1.43, m	19.6
13	1.00, t (6.61)	12.2	0.99, m, overlap	12.4	0.97, t (7.55), overlap	10.5
15	-	161.8	-	161.0	-	162.2

**Table 2:** ^1^H and ^13^C NMR spectroscopic data for compounds **1**–**3** (600 MHz, acetone-*d*_6_).

Position	1		2		3	
	*δ*_H_, mult (*J* in Hz)	*δ*_C_	*δ*_H_, mult (*J* in Hz)	*δ*_C_	*δ*_H_, mult (*J* in Hz)	*δ*_C_
2	3.15, q (6.10, 11.31)	52.1	3.31, brs	50.6	3.24, m	53.7
3	1.61, qd (4.57, 6.10)	42.3	1.69, td (3.74, 7.87)	43.8	1.66, qd (4.17, 6.72)	43.7
4	5.08, overlap	46.9	4.71, dd (3.74)	49.9	4.78, t (7.82)	49.9
4a	-	123.1	-	121.3	-	123.6
5	7.13, d (7.50)	128.8	7.10, d (1.39)	131.7	7.07, d, (7.60)	129.4
6	6.47, td (1.02, 7.43)	116.4	6.52, td (1.39, 7.78)	117.1	6.50, m	117.0
7	6.89, m	128.3	6.92, m	128.5	6.89, m	128.2
8	6.52, d (7.43)	114.2	6.54, d (7.78)	114.8	6.53, d (8.12)	114.6
8a	-	145.1	-	146.0	-	145.7
9	1.54, m	37.8	1.51, m	34.8	1.62/1.55, m	37.0
10	1.54/1.45, m	18.9	1.43, m	18.9	1.43, m	19.2
11	0.93, t (7.18)	14.5	0.94, m, overlap	14.5	0.92, m, overlap	14.5
12	1.32, m	20.3	1.51/1.38, m	20.1	1.50, m	23.0
13	0.98, t (7.42)	12.2	0.96, m, overlap	12.4	0.95, m, overlap	10.4
15	-	159.1	-	158.2	-	159.5
NH-1	5.08, m, overlap	-	4.96, s	-	4.88, s	-
NH-14	5.64, d (9.74)	-	5.74, d (6.69)	-	5.59, d (7.78)	-
NH_2_-16	5.08, m, overlap	-	5.01, s	-	5.09, s	-

## Supplementary Materials

Supplementary materials can be found at https://www.mdpi.com/1422-0067/21/10/3406/s1.
