# Supplementary material for: Protective Effect of Tetrahydroquinolines from the Edible Insect Allomyrina dichotoma on LPS-Induced Vascular Inflammatory Responses
Source: Int J Mol Sci. 2020 May 12;21(10):3406. doi: 10.3390/ijms21103406 (PMC7279263; doi:10.3390/ijms21103406)
Supplement: Supplementary file 1 [file ijms-21-03406-s001.pdf]

Supplementary Materials

Tetrahydroquinolines from the edible insect *Allomyrina dichotoma* modulate vascular inflammatory responses in LPS-mediated human endothelial cells

InWha Park [1,†], Wonhwa Lee [2,†], Youngbum Yoo [2], Hyosoo Shin [2], Joonseok Oh [3], Hyelim Kim [1], Mi-Ae Kim [4], Jae Sam Hwang [4], Jong-Sup Bae [5,*], and MinKyun Na [1,*]

[1] College of Pharmacy, Chungnam National University, Daejeon 34134, Republic of Korea; inwha129@naver.com (I.P.); rimeeyo@gmail.com (H.K.)

[2] Aging Research Center, Korea Research Institute of Bioscience and Biotechnology (KRIBB), Daejeon, Republic of Korea; wonhwalee@kribb.re.kr (W.L.); ddq3416@gmail.com (Y.Y.); hysooo0914@gmail.com (H.S.)

[3] Department of Chemistry, Yale University, New Haven, Connecticut 06520, United States; joonseok.oh@yale.edu (J.O.)

[4] Department of Agricultural Biology, The National Academy of Agricultural Science, RDA, Wanju-gun 55365, Republic of Korea; kimma@korea.kr (M.A.K.); hwangjs@korea.kr (J.S.H.)

[5] College of Pharmacy, CMRI, Research Institute of Pharmaceutical Sciences, BK21 Plus KNU Multi-Omics based Creative Drug Research Team, Kyungpook National University, Daegu 41566, Republic of Korea

* Correspondence: mkna@cnu.ac.kr (M.N.); baejs@knu.ac.kr (J.S.B.); Tel.: +82-42-821-5925 (M.N.); Tel.: +82-53-950-8570 (J.S.B.)

Index

Figure S1. Purity evaluation of compound **1**······ S4

Figure S2. HRESIMS spectrum of compound **1**······ S4

Figure S3. ^{1}H NMR spectrum of compound **1** in methanol-d_4······ S5

Figure S4. ^{13}C NMR spectrum of compound **1** in methanol-d_4······S5

Figure S5. ^{1}H NMR spectrum of compound **1** in acetone-d_6······ S6

Figure S6. ^{13}C NMR spectrum of compound **1** in acetone-d_6······ S6

Figure S7. HSQC spectrum of compound **1** in methanol-d_4······ S7

Figure S8. HMBC spectrum of compound **1** in methanol-d_4······ S7

Figure S9. COSY spectrum of compound **1** in methanol-d_4······ S8

Figure S10. NOESY spectrum of compound **1** in methanol-d_4······ S8

Figure S11. Purity evaluation of compound **2**······ S9

Figure S12. HRESIMS spectrum of compound **2**······ S9

Figure S13. ^{1}H NMR spectrum of compound **2** in methanol-d_4······ S10

Figure S14. ^{13}C NMR spectrum of compound **2** in methanol-d_4······ S10

Figure S15. ^{1}H NMR spectrum of compound **2** in acetone-d_6······ S11

Figure S16. ^{13}C NMR spectrum of compound **2** in acetone-d_6······ S11

Figure S17. HSQC spectrum of compound **2** in acetone-d_6······ S12

Figure S18. HMBC spectrum of compound **2** in acetone-d_6······ S12

Figure S19. COSY spectrum of compound **2** in acetone-d_6······ S13

Figure S20. NOESY spectrum of compound **2** in acetone-d_6······ S13

Figure S21. Purity evaluation of compound **3**······ S14

Figure S22. HRESIMS spectrum of compound **3**······ S14

Figure S23. ^{1}H NMR spectrum of compound **3** in methanol-d_4······ S15

Figure S24. ^{13}C NMR spectrum of compound **3** in acetone-d_6······ S15

Figure S25. ^{1}H NMR spectrum of compound **3** in acetone-d_6······ S16

Figure S26. ^{13}C NMR spectrum of compound **3** in acetone-d_6······ S16

Figure S27. HSQC spectrum of compound **3** in acetone-d_6······ S17

Figure S28. HMBC spectrum of compound **3** in acetone-d_6······ S17

Figure S29. COSY spectrum of compound **3** in acetone-d_6······ S18

Figure S30. NOESY spectrum of compound **3** in acetone-d_6 ··· S18

Figure S31. Candidate diastereomers **3a** and **3b** ··· S19

Figure S32. Results of DP4+ analysis of compound **3** ··· S22

Figure S33. Purity evaluation of **4** based on HPLC-ELSD application ··· S23

Figure S34. HRESIMS spectrum of compound **4** ··· S23

Figure S35. ^{1}H NMR spectrum of compound **4** in DMSO-d_6 ··· S24

Figure S36. ^{13}C NMR spectrum of compound **4** in DMSO-d_6 ··· S24

Figure S37. HSQC spectrum of compound **4** in DMSO-d_6 ··· S25

Figure S38. HMBC spectrum of compound **4** in DMSO-d_6 ··· S25

Figure S39. Key HMBC correlations of compound **4** ··· S26

Figure S40. ^{1}H NMR spectrum of compound **5** in DMSO-d_6 ··· S27

Figure S41. ^{1}H NMR spectrum of compound **6** in methanol-d_4 ··· S27

Figure S42. ^{1}H NMR spectrum of compound **7** in DMSO-d_6 ··· S28

Figure S43. ^{1}H NMR spectrum of compound **8** in methanol-d_4 ··· S28

Figure S44. Calibration curve of compounds **1**-**3** in crude extract ··· S29

Table S1. The major conformers of diastereomers of compound **3** ··· S20

Table S2. Experimental and calculated ^{1}H chemical shift value of **3** and its possible diastereomers **3a** and **3b**, repectively ··· S21

Table S3. Experimental and calculated ^{13}C chemical shift values of **3** and its possible diastereomers **3a** and **3b**, respectively ··· S21

Materials and methods of biological activities ··· S30

Reference ··· S36

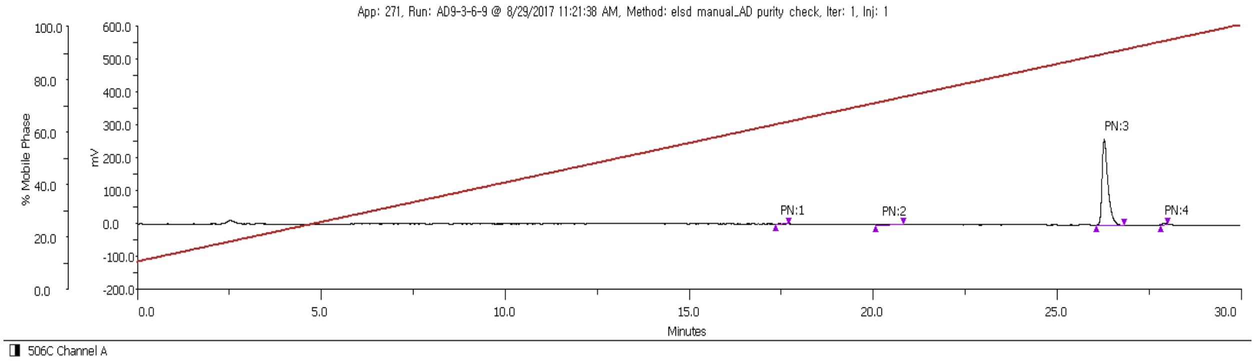

Sample Table

Injection Number	Peak Name	Retention Time (min)	Area (uVmin x100)	Height (mV)	Sample Name	Sample Location	Fraction Site(s)	Area %	
1	1	17.476	14490.2445	1.303	Sample			0.29	
1	2	20.23	48472.1712	1.528	Sample			0.969	
1	3	26.304	4895013.4115	262.489	Sample			97.806	
1	4	27.921	46860.4688	3.604	Sample			0.936	

Purity (%) > 97 %

Figure S1. Purity evaluation of 1 based on HPLC-ELSD application

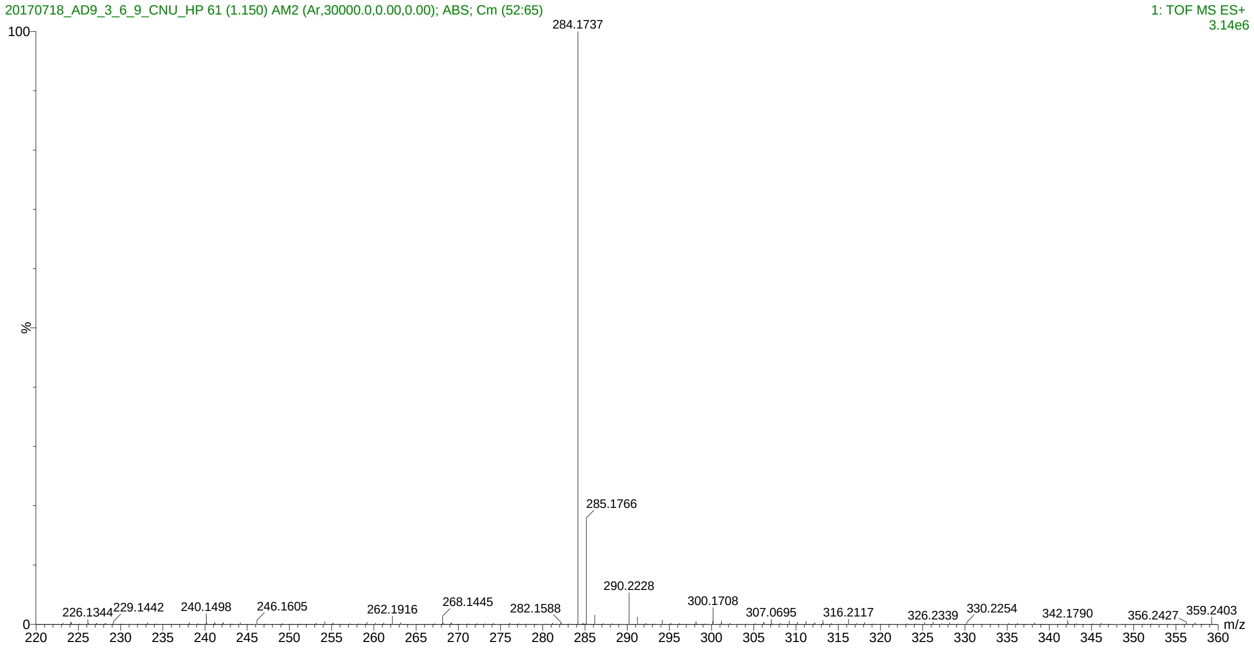

Elemental Composition Report

Mass	Calc. Mass	mDa	PPM	DBE	i-FIT		Norm	Conf(%) Formula
284.1737	284.1739	-0.2	-0.7	5.5	1064.7	n/a	n/a	C15 H23 N3 O Na

Figure S2. HRESIMS data of compound 1

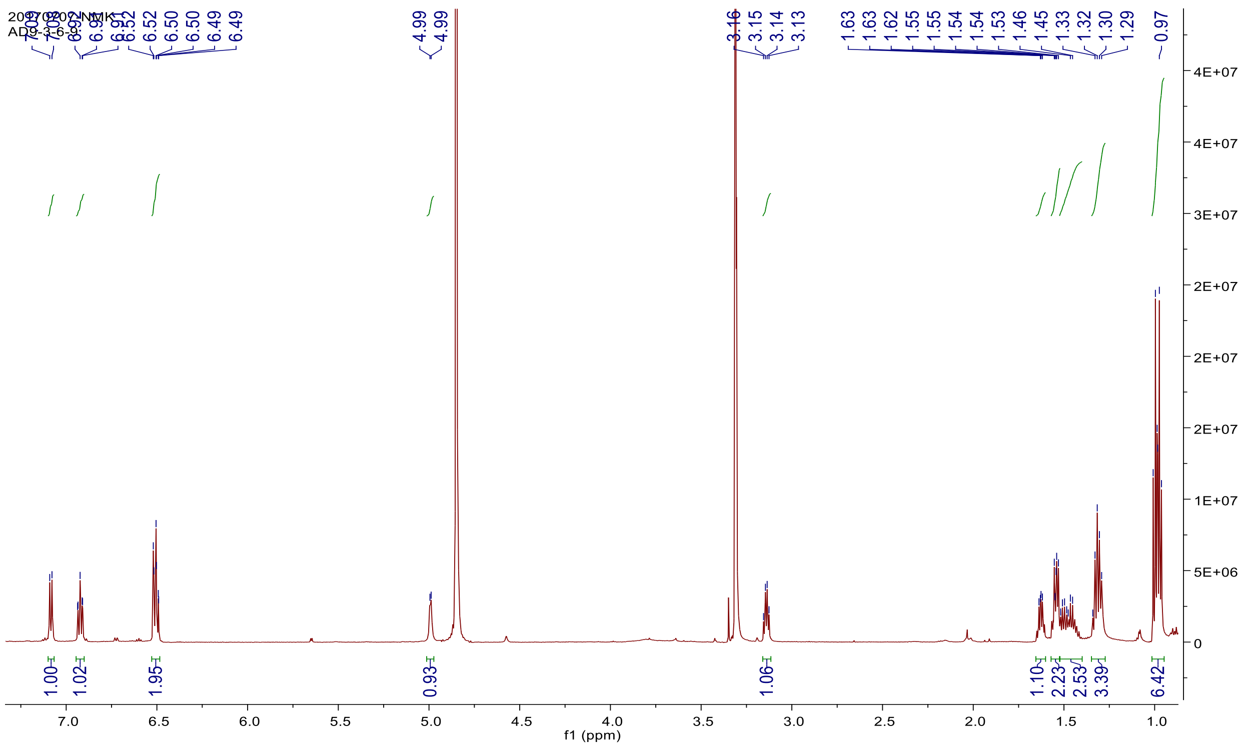

Figure S3. ^{1}H NMR spectrum of compound 1 in methanol-*d*4 (600 MHz)

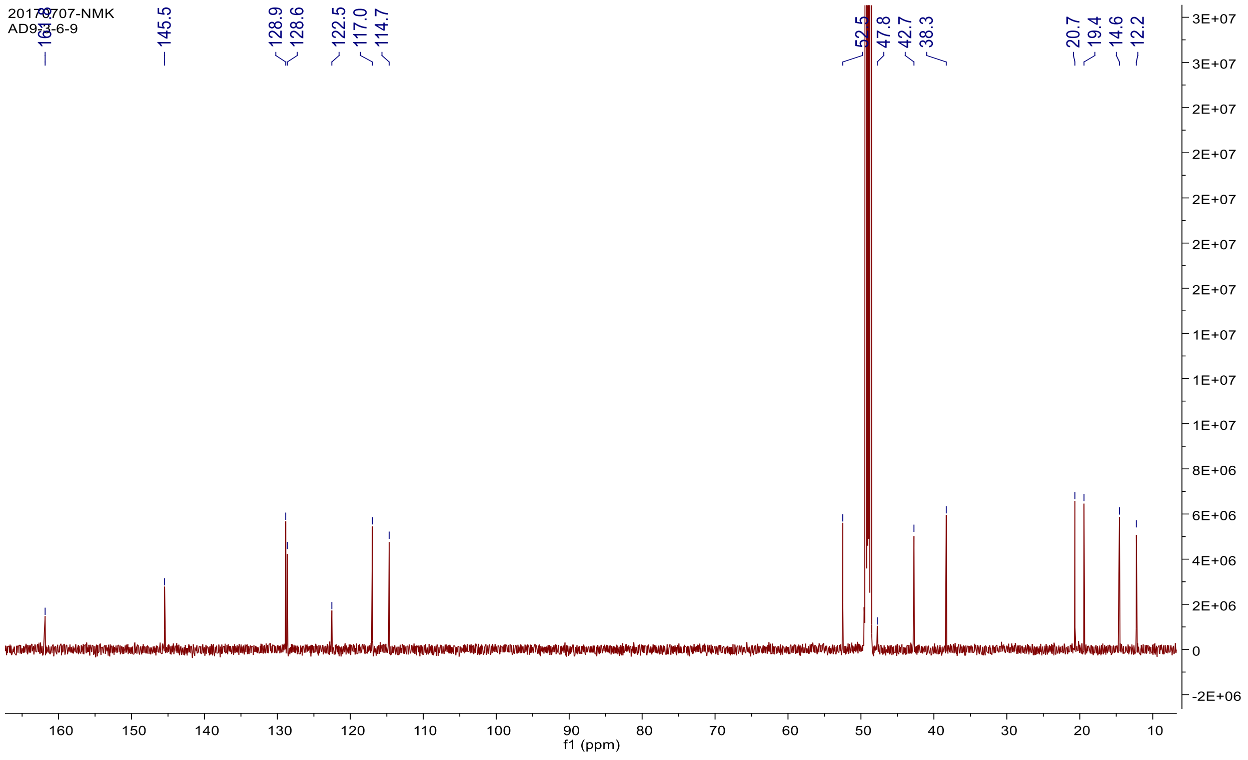

Figure S4. ^{13}C NMR spectrum of compound 1 in methanol-*d*4 (150 MHz)

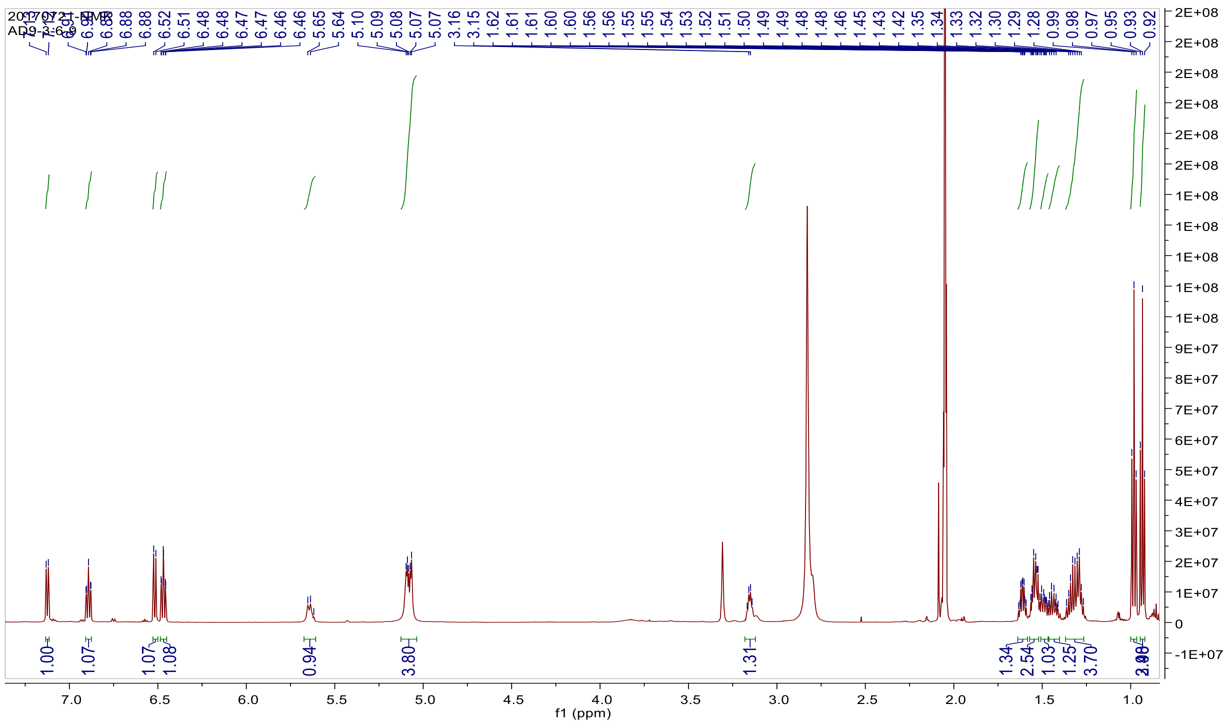

Figure S5. ^{1}H NMR spectrum of compound 1 in acetone-d_6 (600 MHz)

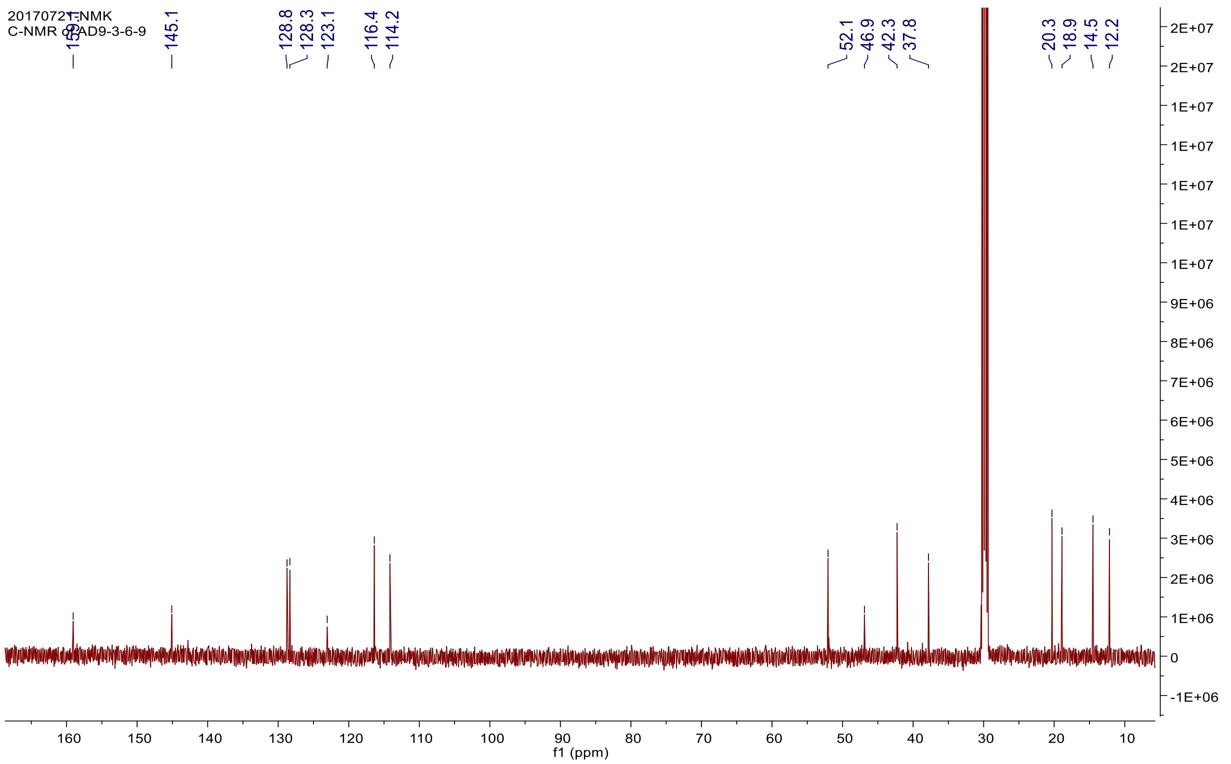

Figure S6. ^{13}C NMR spectrum of compound 1 in acetone-d_6 (150 MHz)

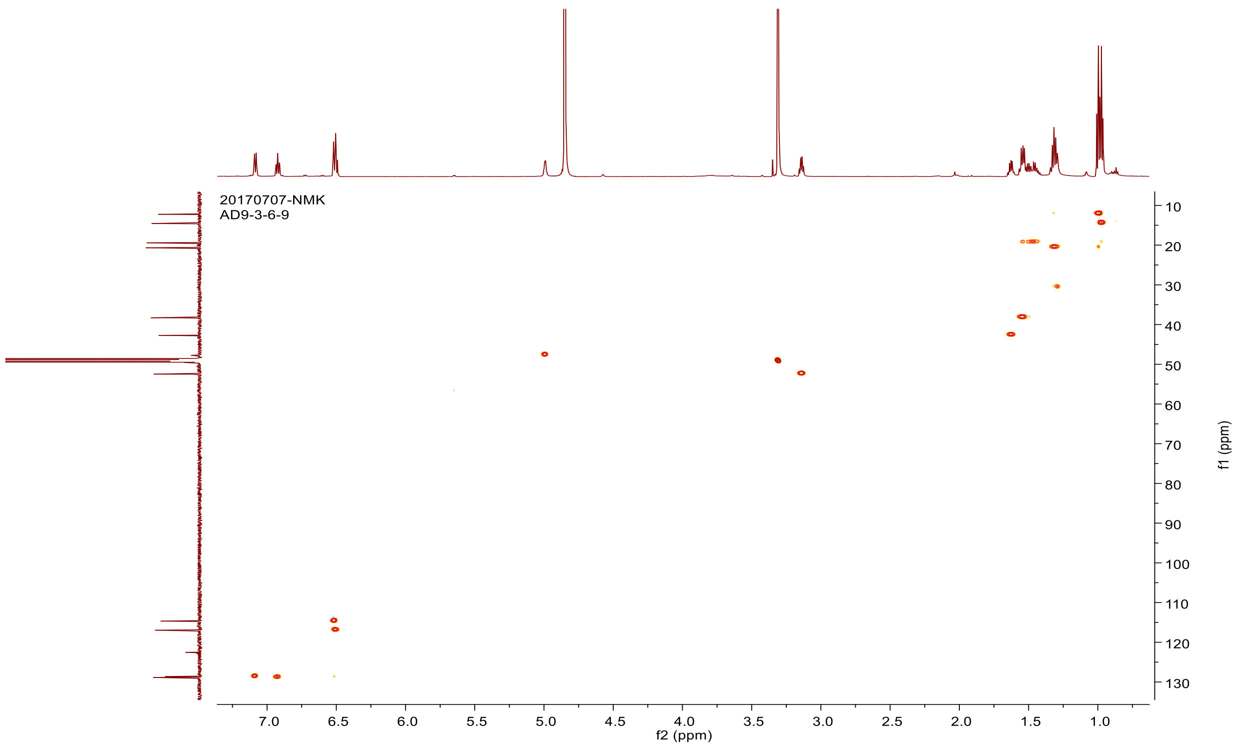

Figure S7. HSQC spectrum of compound 1 in methanol-*d*4 (600 MHz)

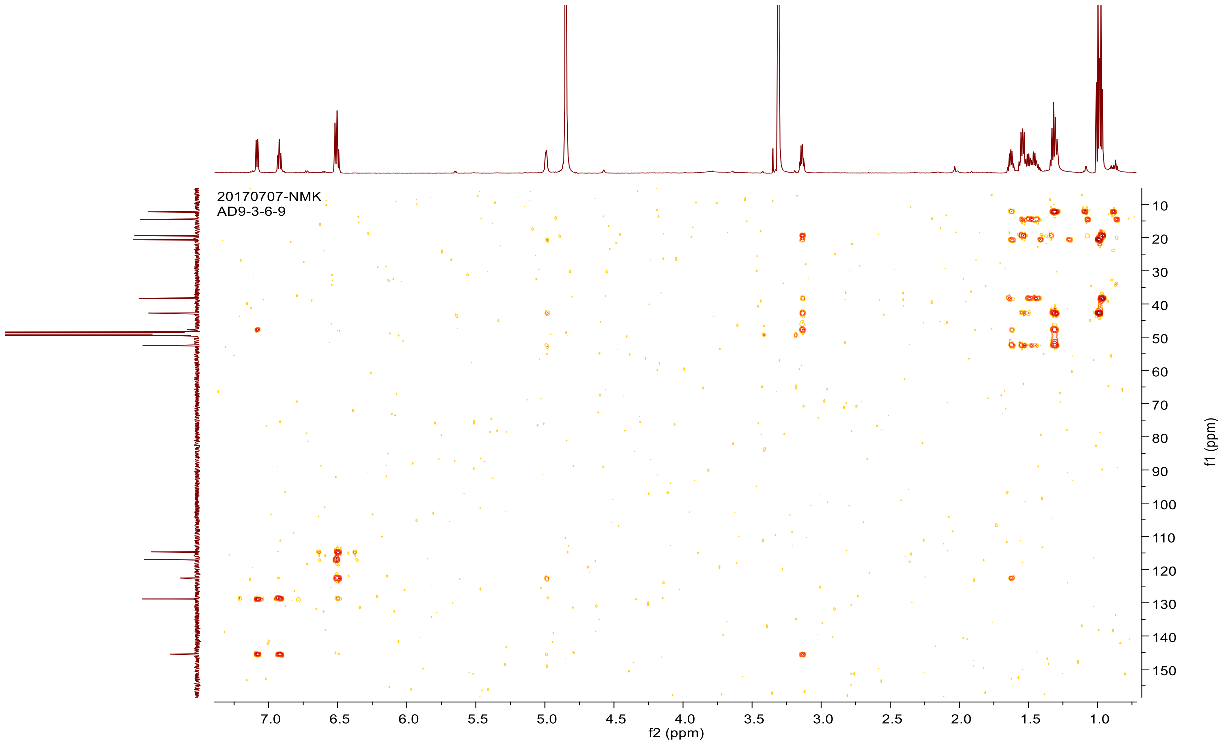

Figure S8. HMBC spectrum of compound 1 in methanol-*d*4 (600 MHz)

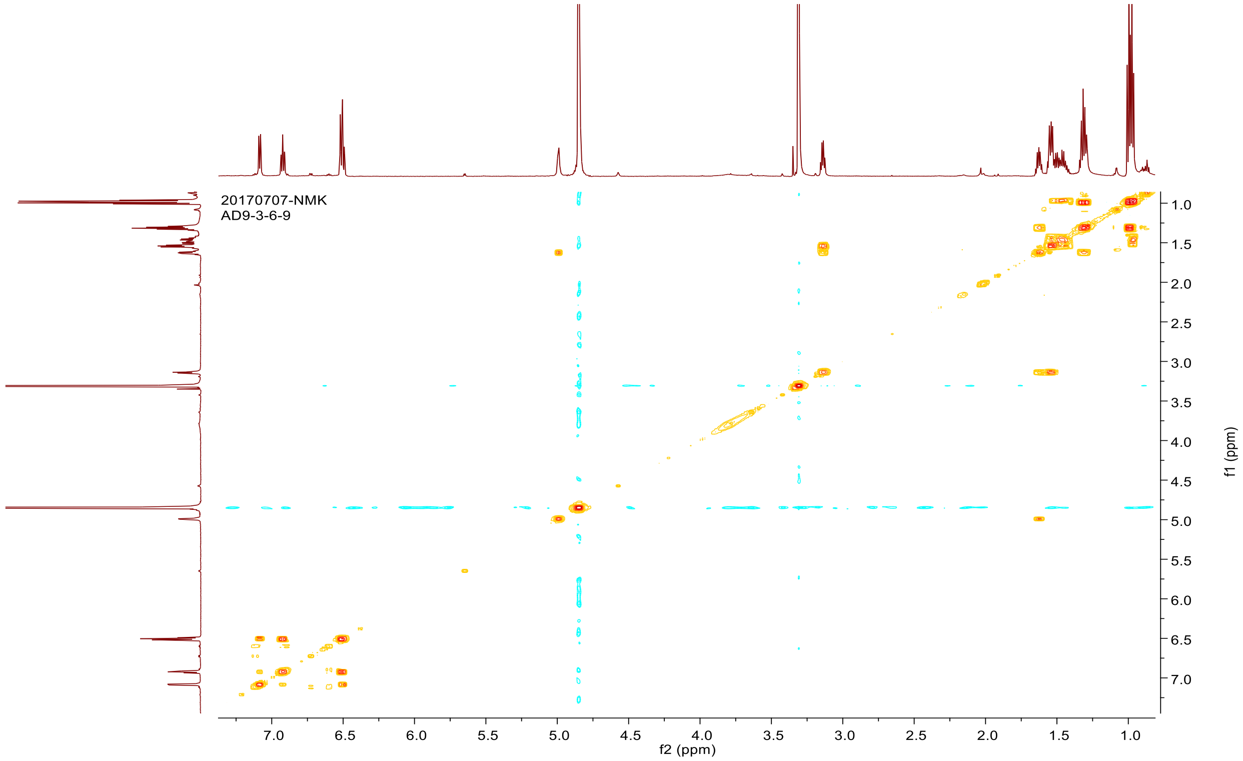

Figure S9. COSY data of compound 1 in methanol-*d*₄ (600 MHz)

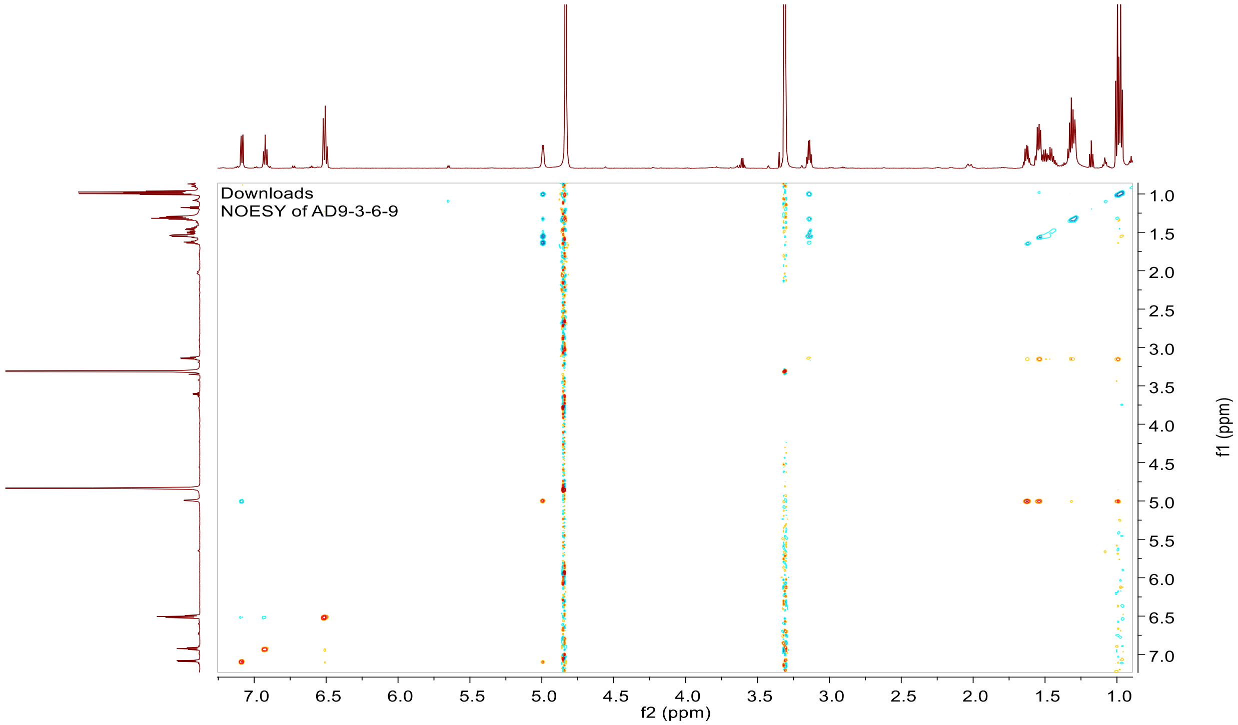

Figure S10. NOESY data of compound 1 in methanol-*d*₄ (600 MHz)

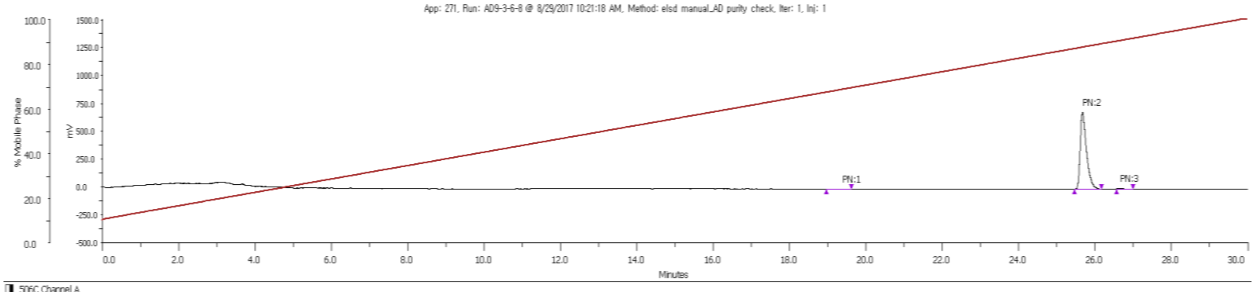

Sample Table

Injection Number	Peak Name	Retention Time (min)	Area (uV*min x100)	Height (mV)	Sample Name	Sample Location	Fraction Site(s)	Area %	
1	1	19.427	119771.2479	3.213	Sample			0.881	
1	2	25.687	13293488.75	686.91	Sample			97.805	
1	3	26.657	178578.3821	9.173	Sample			1.314	

Purity (%) > 97 %

Figure S11. Purity evaluation of 2 based on HPLC-ELSD application

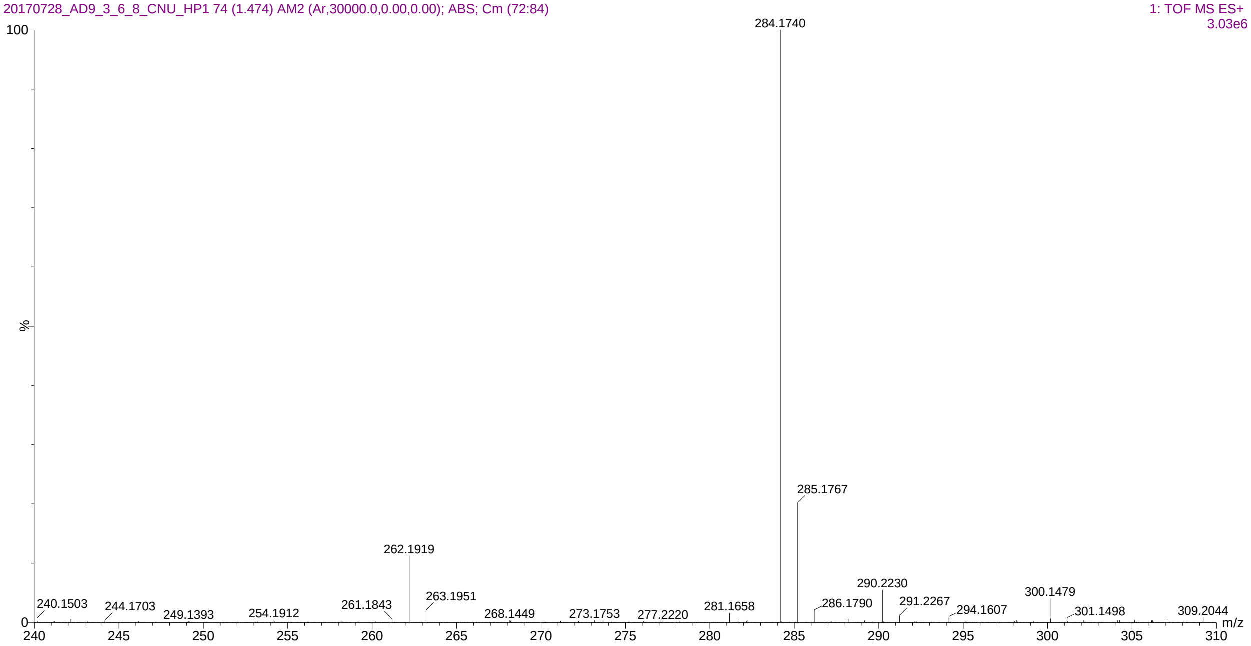

Elemental Composition Report

Mass	Calc. Mass	mDa	PPM	DBE	i-FIT		Norm	Conf(%) Formula
262.1919	262.1919	0.0	0.0	5.5	699.3	n/a	n/a	C15 H24 N3 O
284.1740	284.1739	0.1	0.4	5.5	982.1	n/a	n/a	C15 H23 N3 O Na

Figure S12. HRESIMS spectrum of compound 2

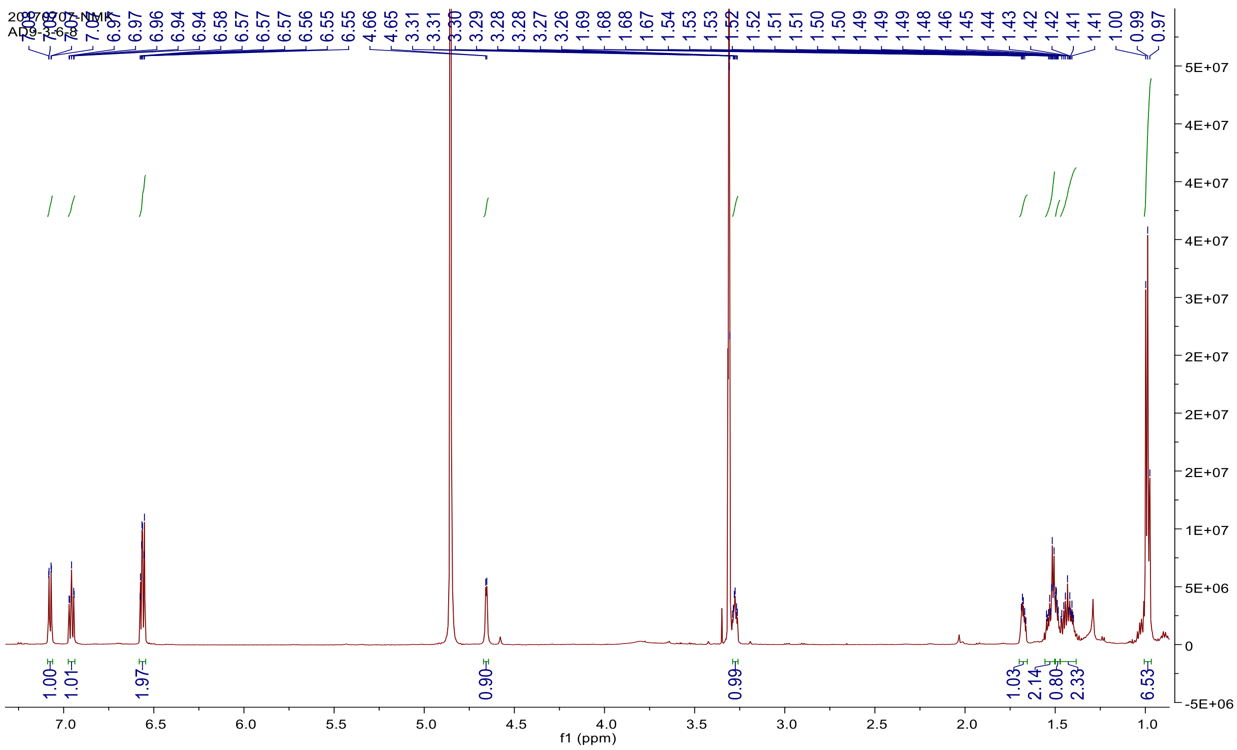

Figure S13. ^{1}H NMR spectrum of compound 2 in methanol-d_4 (600 MHz)

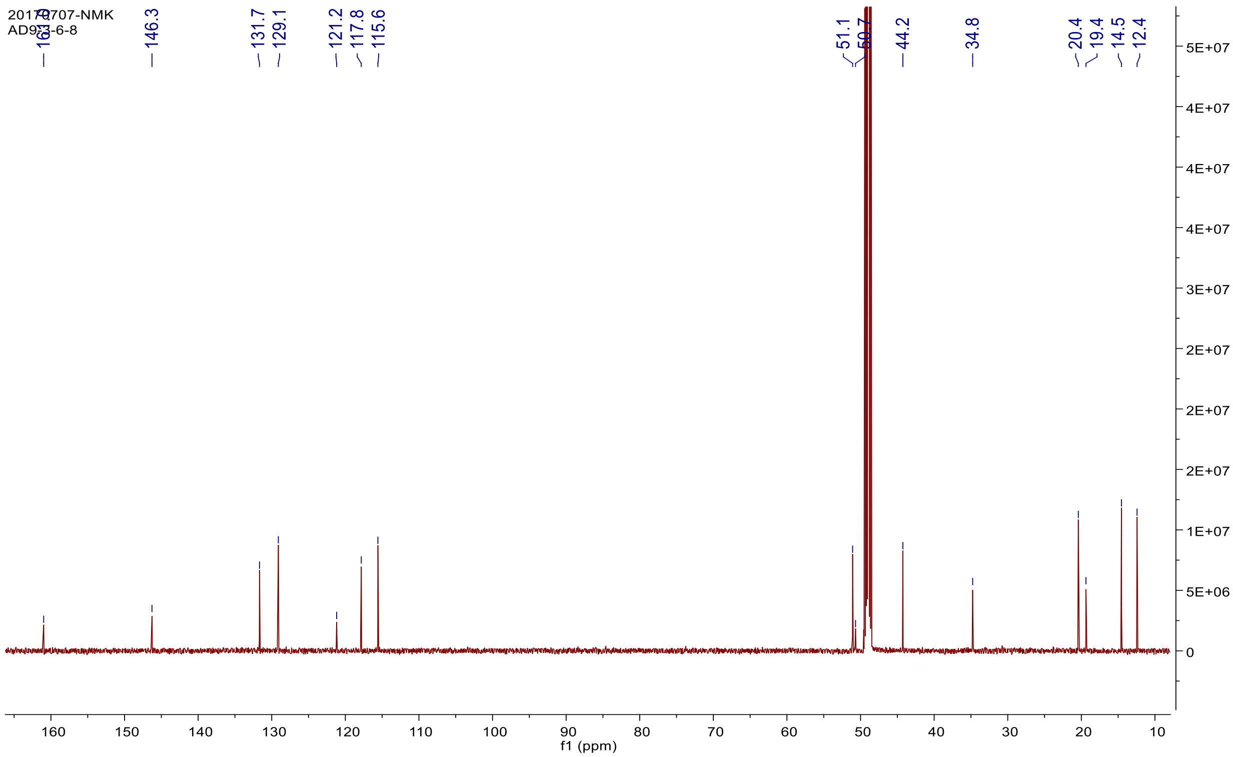

Figure S14. ^{13}C NMR spectrum of compound 2 in methanol-d_4 (150 MHz)

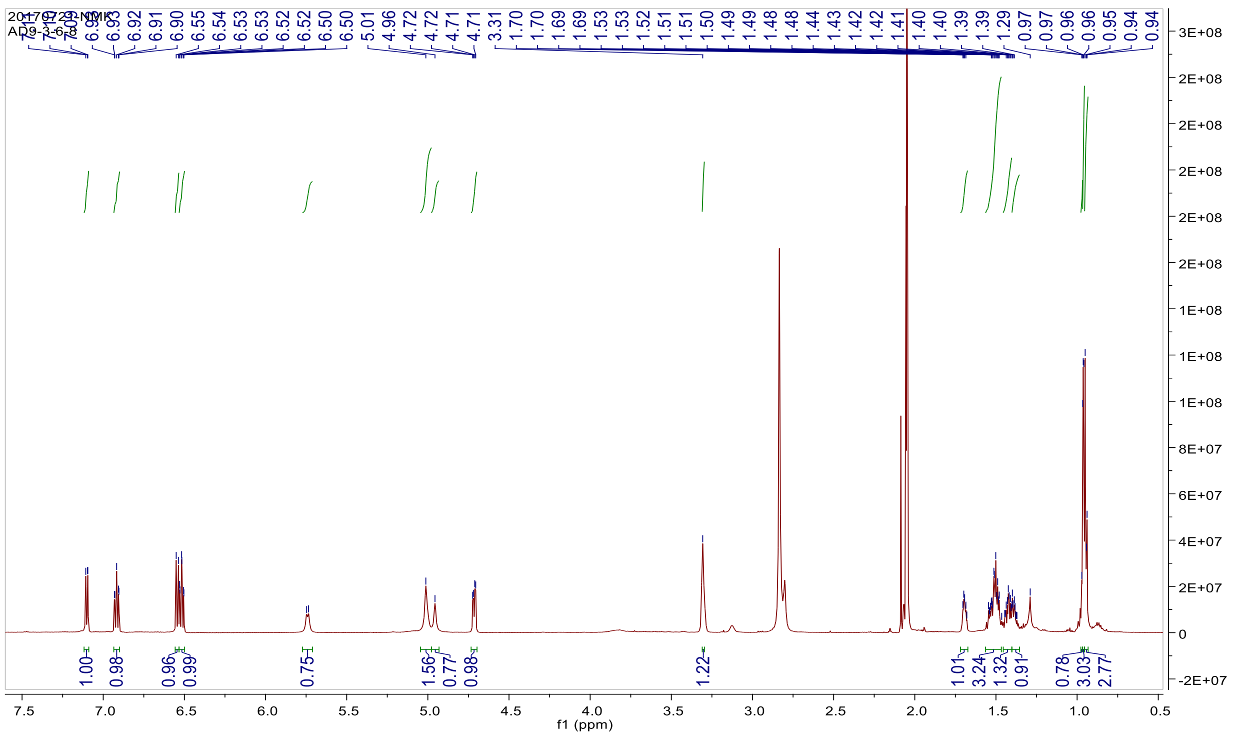

Figure S15. ^{1}H NMR spectrum of compound 2 in acetone-d_6 (600 MHz)

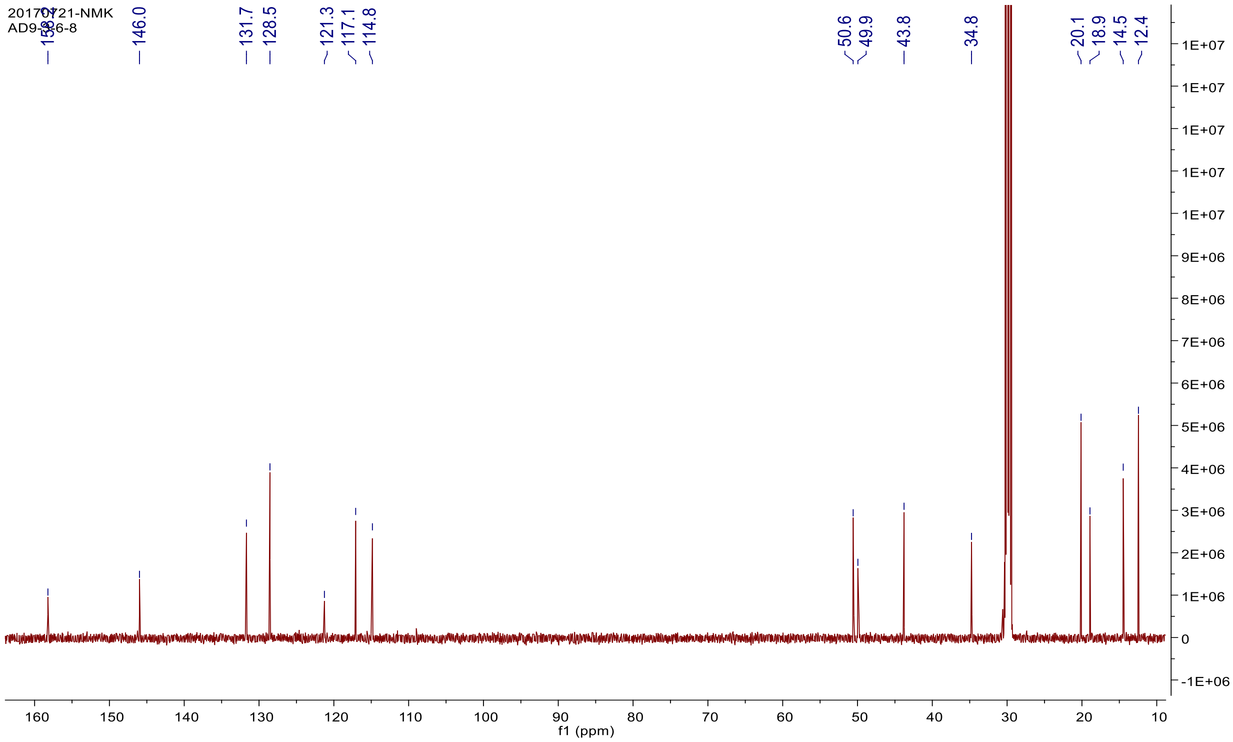

Figure S16. ^{13}C NMR spectrum of compound 2 in acetone-d_6 (150 MHz)

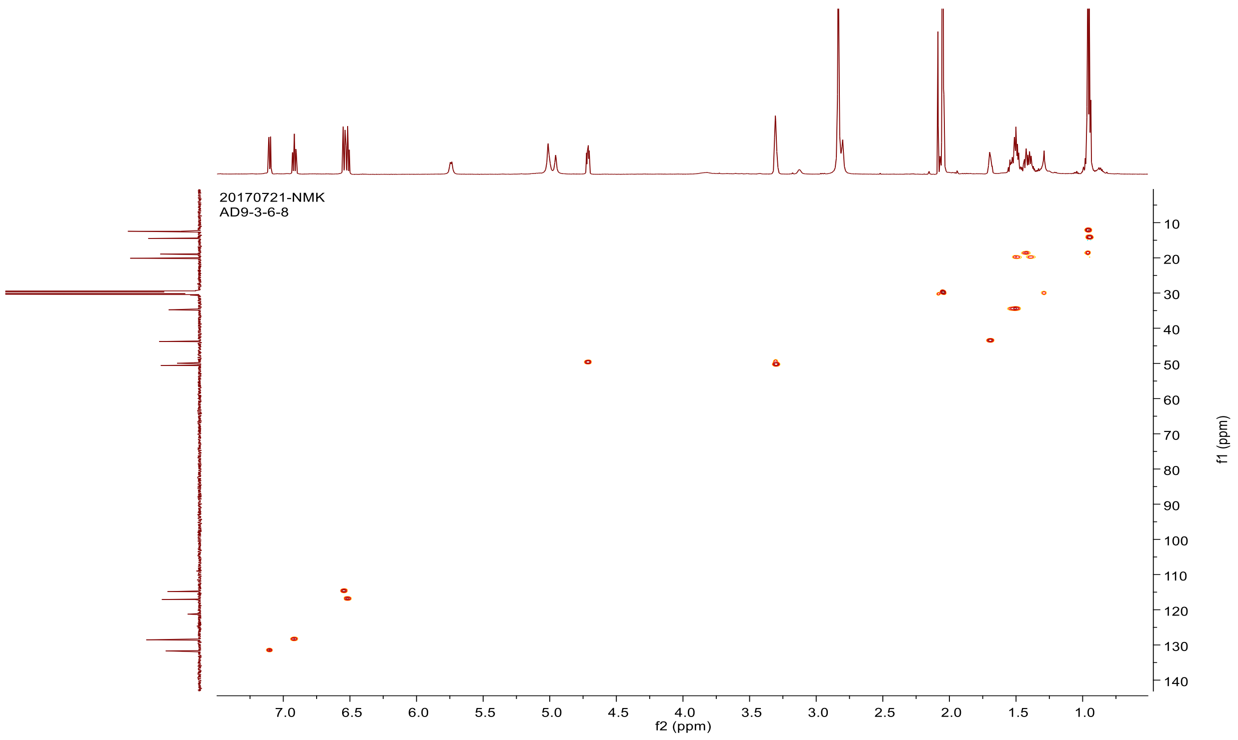

Figure S17. HSQC spectrum of compound 2 in acetone-d_6 (600 MHz)

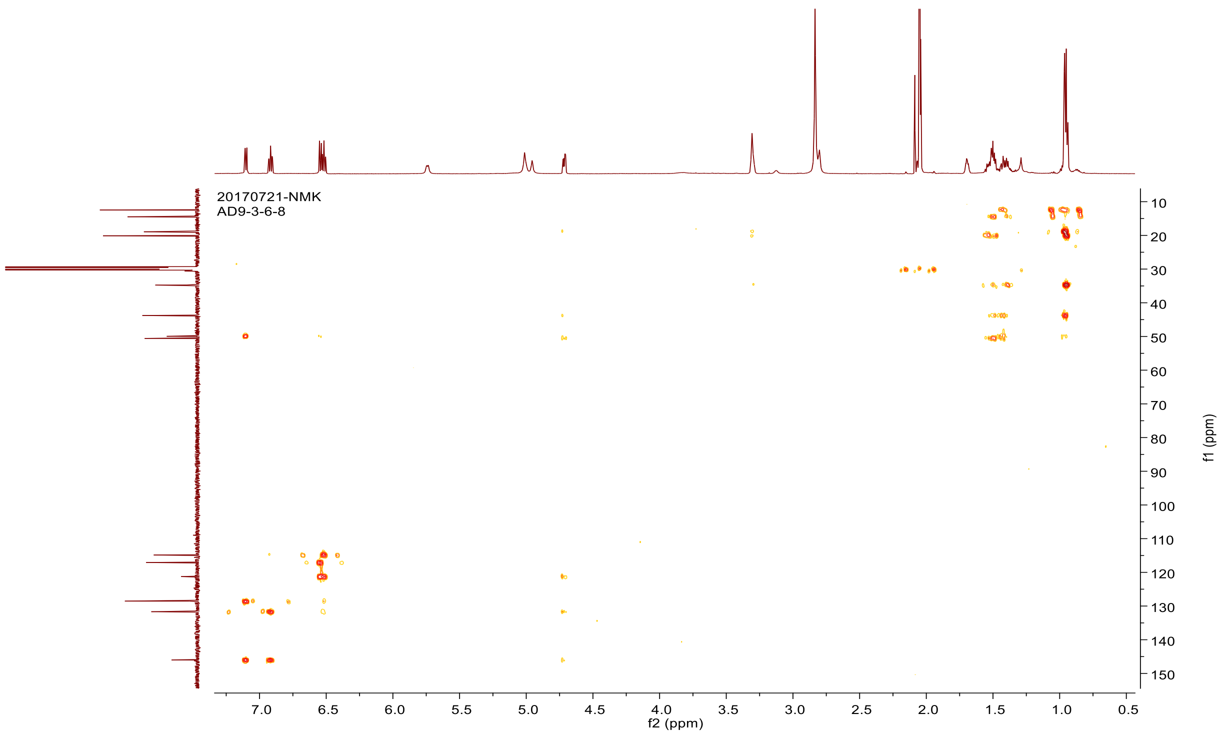

Figure S18. HMBC spectrum of compound 2 in acetone-d_6 (600 MHz)

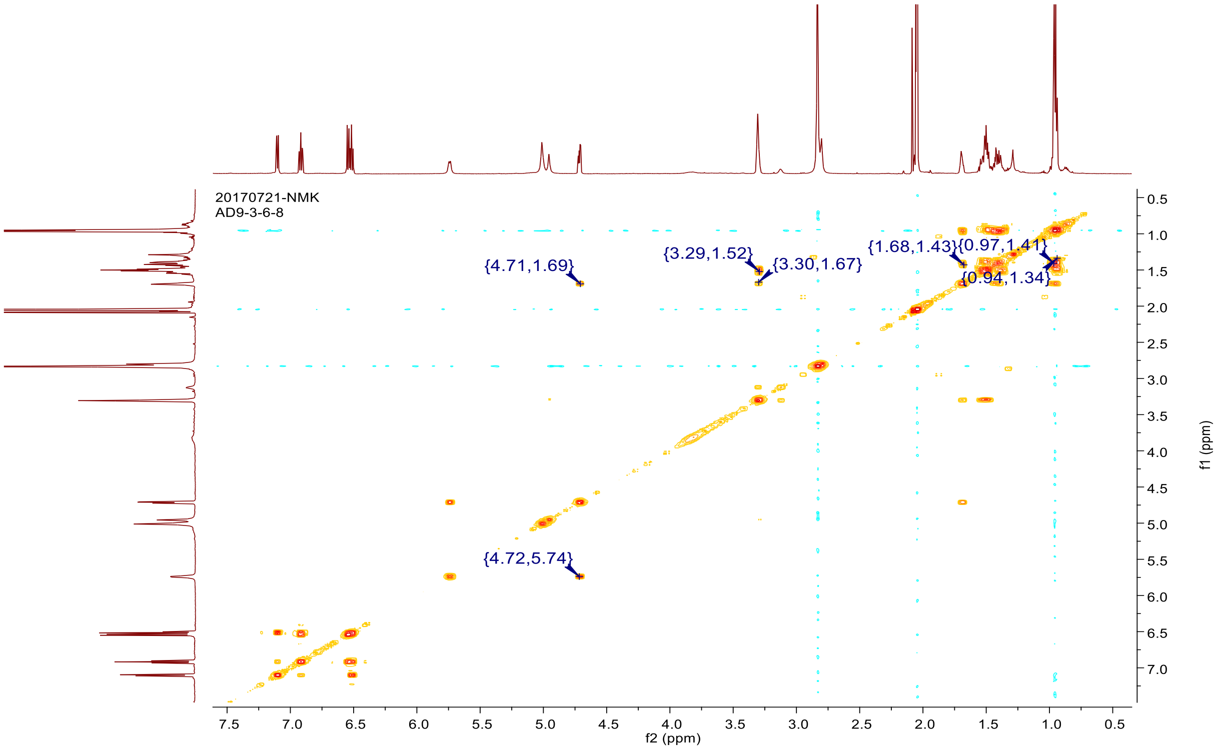

Figure S19. COSY spectrum of compound 2 in acetone-d_6 (600 MHz)

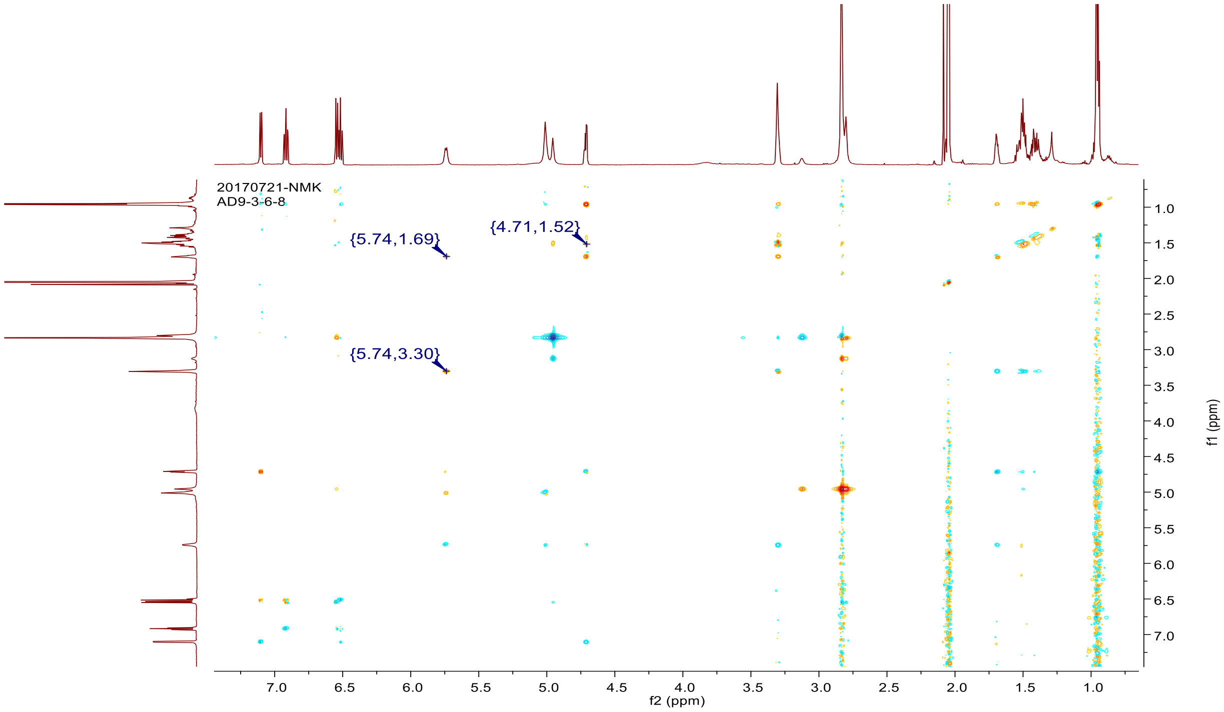

Figure S20. NOESY spectrum of compound 2 in acetone-d_6 (600 MHz)

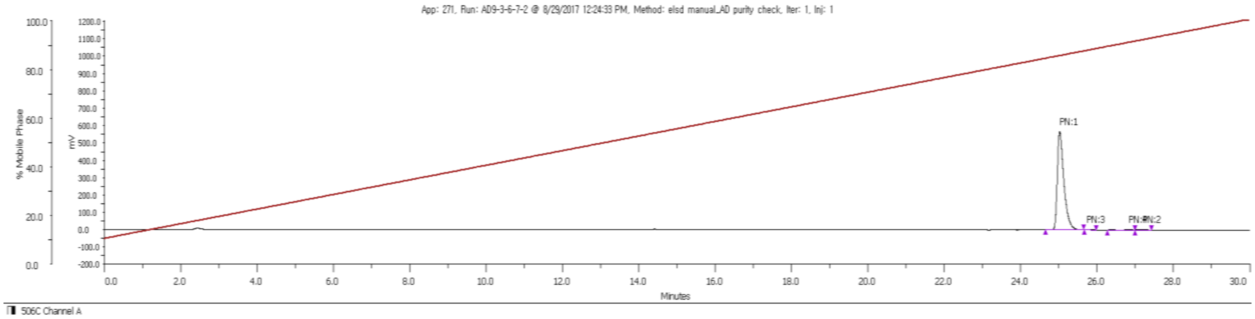

Sample Table

Injection Number	Peak Name	Retention Time (min)	Area (uV*min x100)	Height (mV)	Sample Name	Sample Location	Fraction Site(s)	Area %	
1	1	25.039	11241920.4136	562.439	Sample			98.392	
1	2	27.195	88057.5013	4.257	Sample			0.771	
1	3	25.892	19912.4863	1.023	Sample			0.174	
1	4	26.823	75758.4233	1.995	Sample			0.663	

Purity (%) > 98%

Figure S21. Purity evaluation of 3 based on HPLC-ELSD application

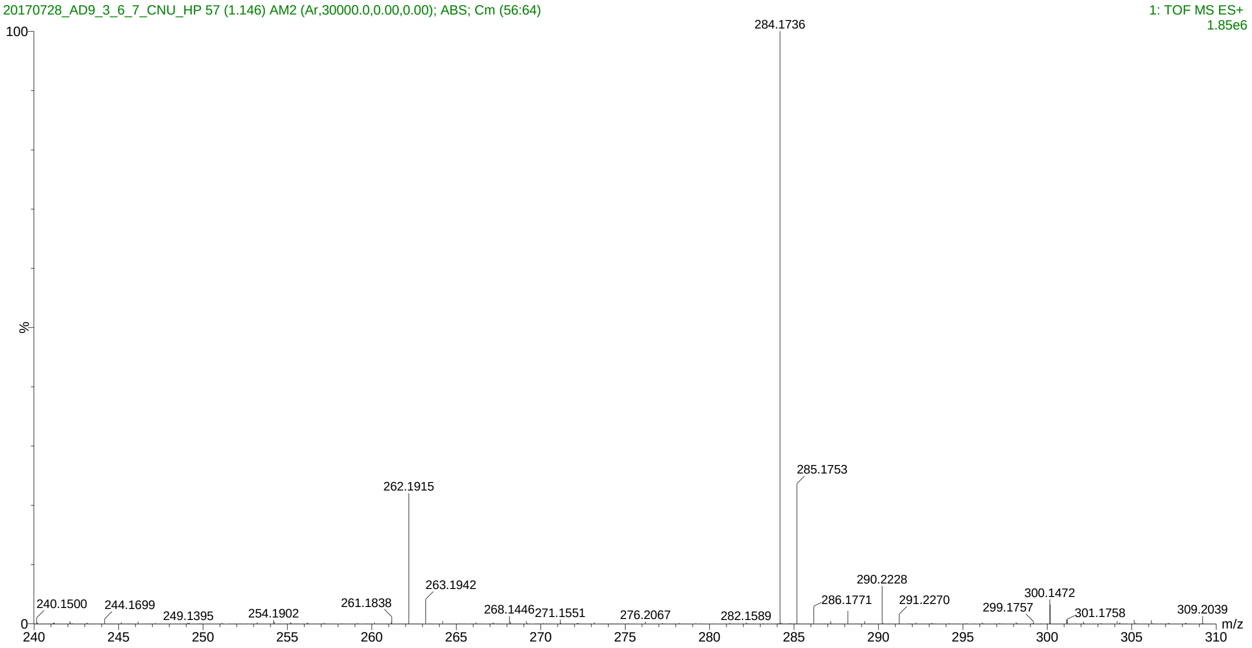

Elemental Composition Report

Mass	Calc. Mass	mDa	PPM	DBE	i-FIT		Norm	Conf(%) Formula
262.1915	262.1919	-0.4	-1.5	5.5	797.2	n/a	n/a	C15 H24 N3 O
284.1736	284.1739	-0.3	-1.1	5.5	1016.4	n/a	n/a	C15 H23 N3 O Na

Figure S22. HRESIMS spectrum of compound 3

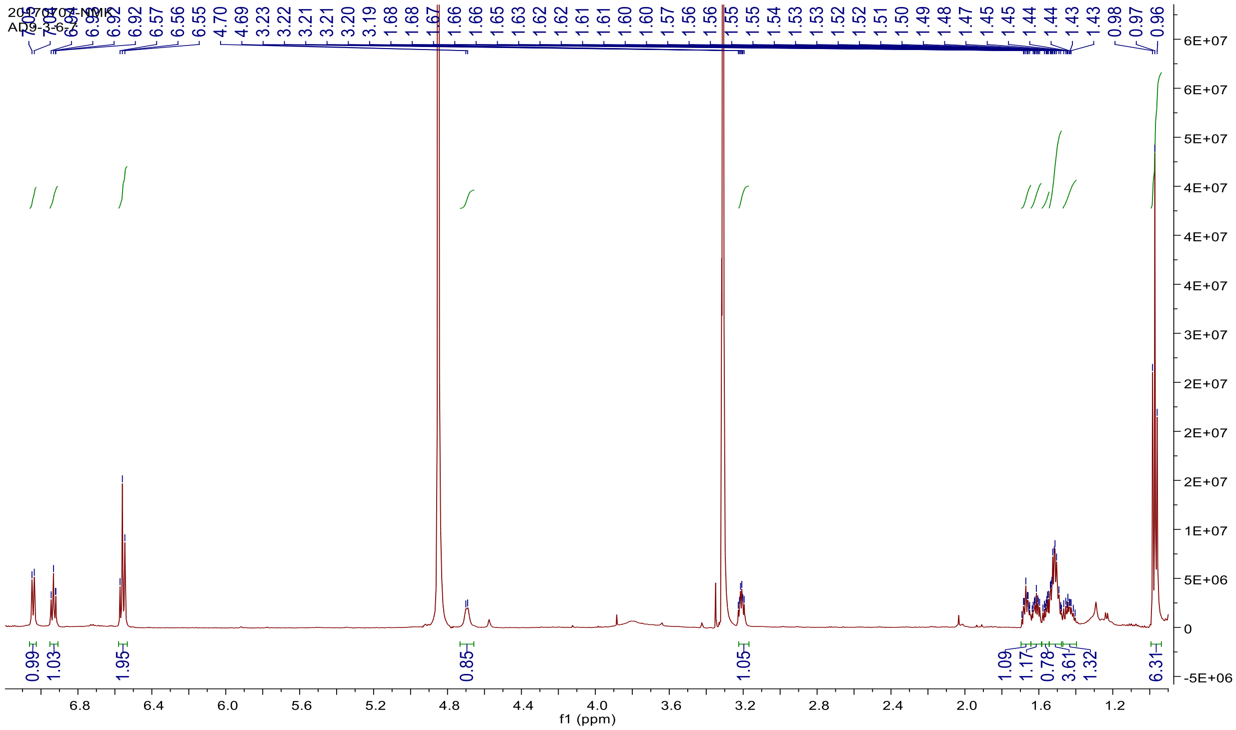

Figure S23. ^{1}H NMR spectrum of compound 3 in methanol-d_4 (600 MHz)

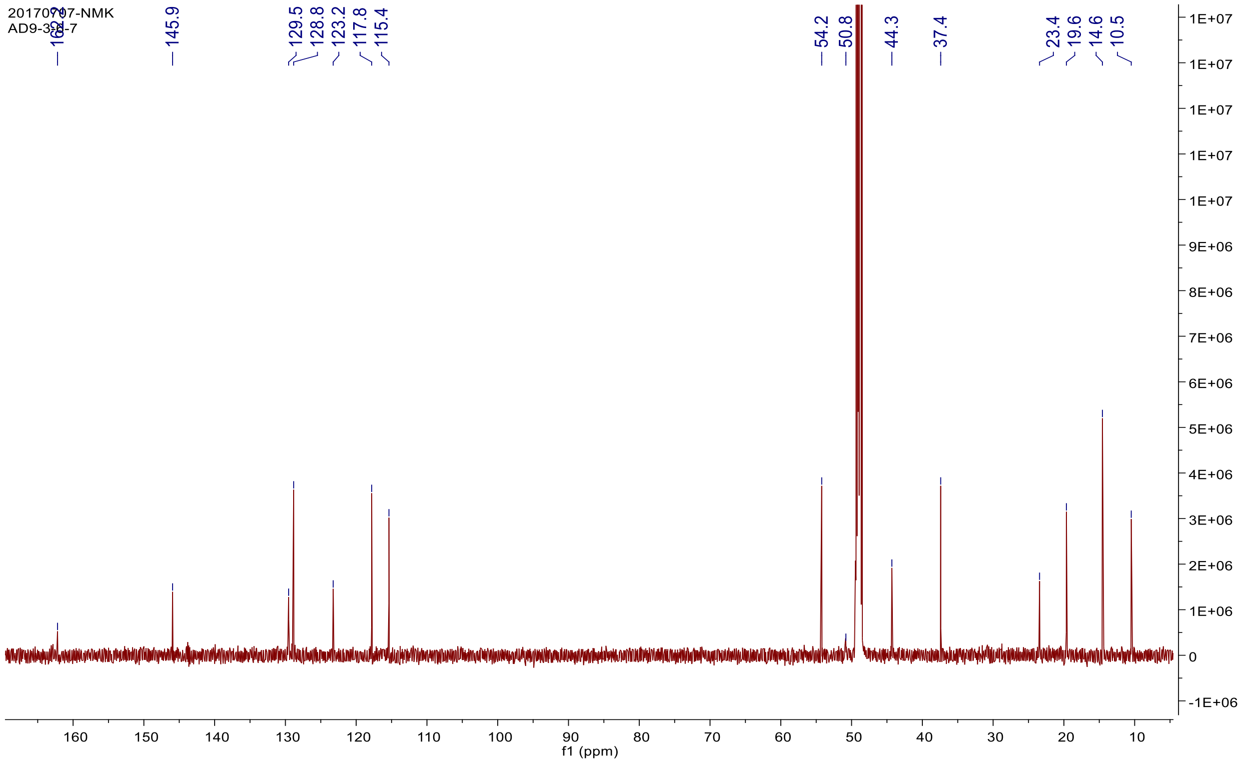

Figure S24. ^{13}C NMR spectrum of compound 3 in methanol- d_4 (150 MHz)

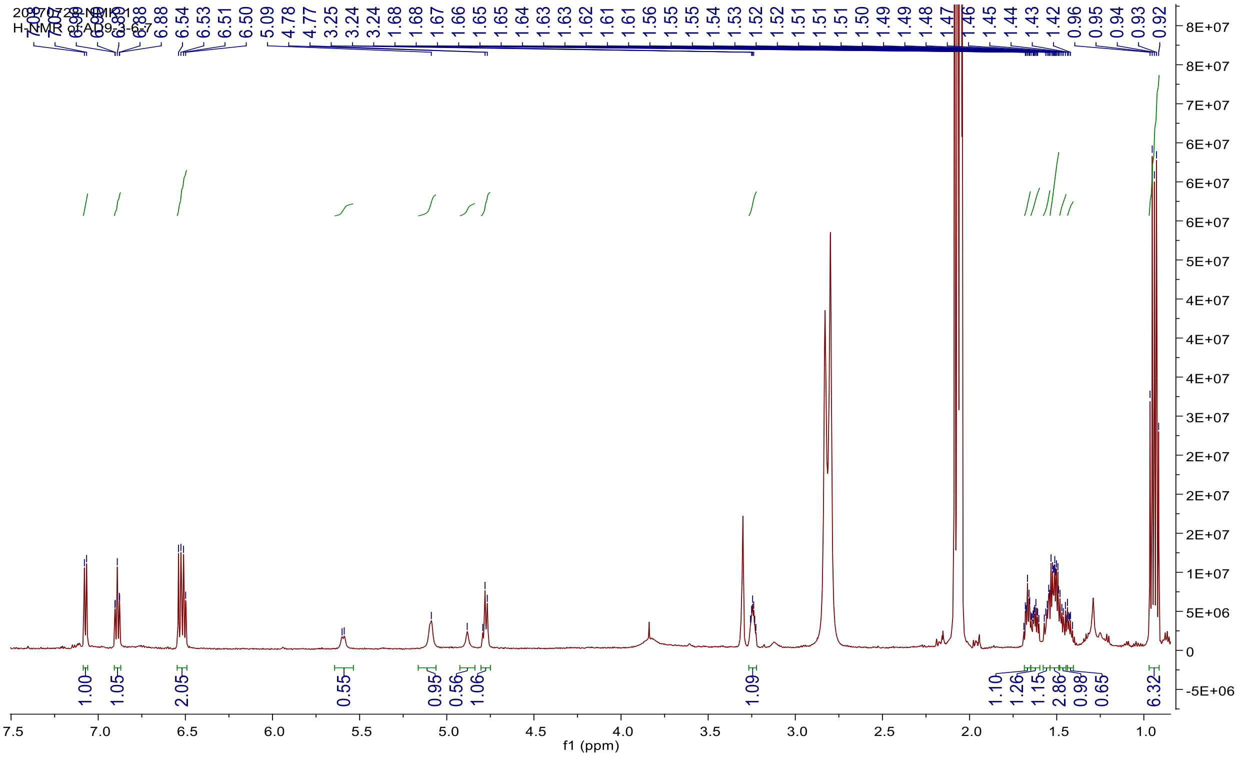

Figure S25. **^{1}H NMR spectrum of compound 3 in acetone-*d*6 (600 MHz)**

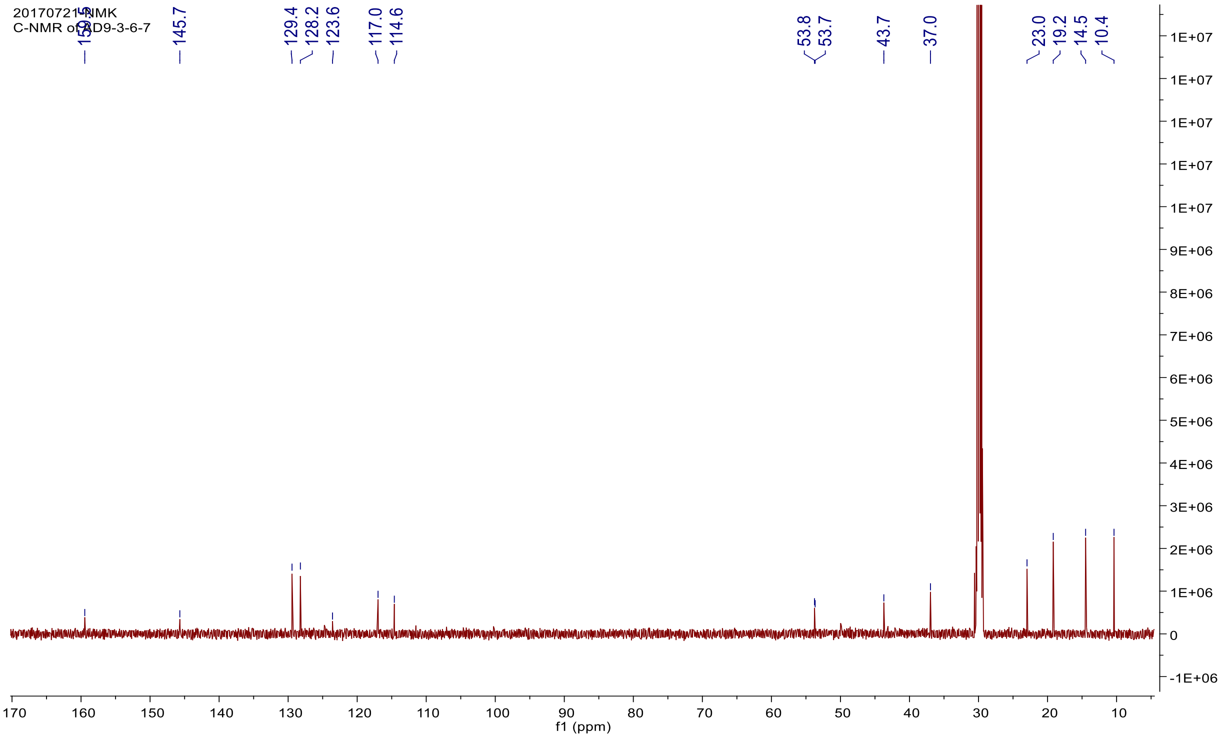

Figure S26. ^{13}C NMR spectrum of compound 3 in acetone-*d*6 (150 MHz)

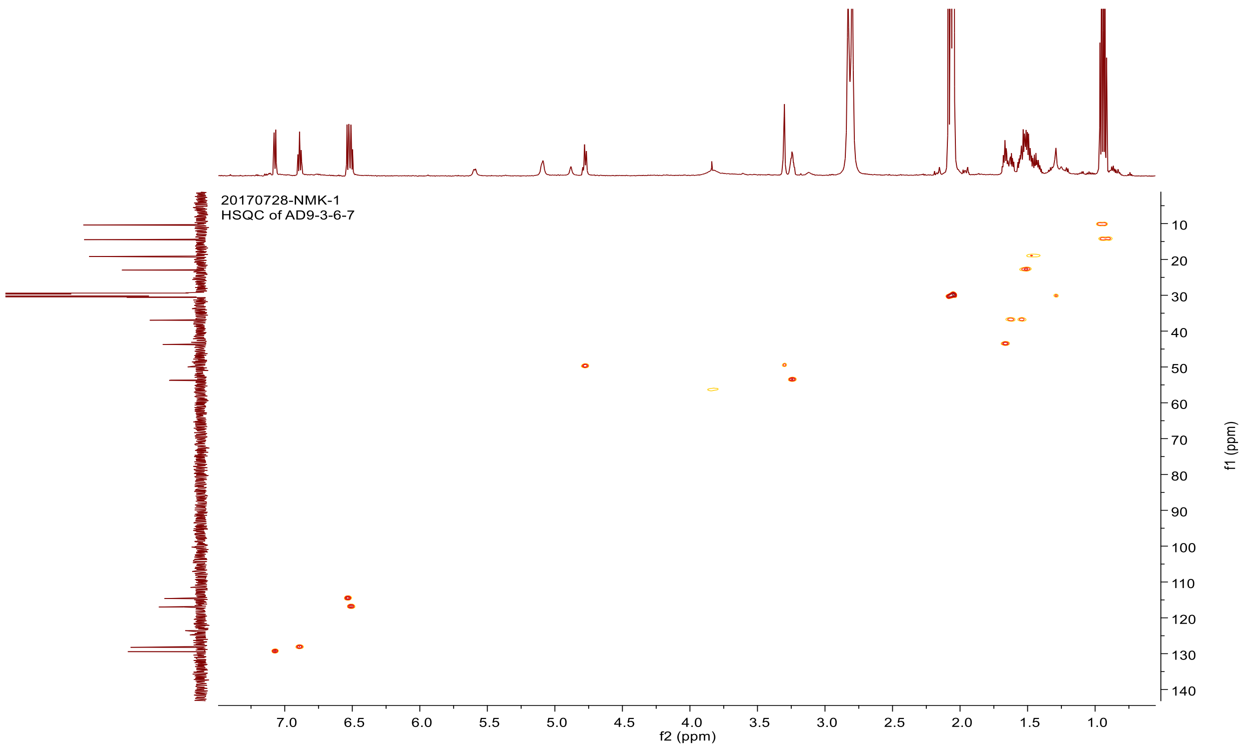

Figure S27. HSQC spectrum of compound 3 in acetone-d_6 (600 MHz)

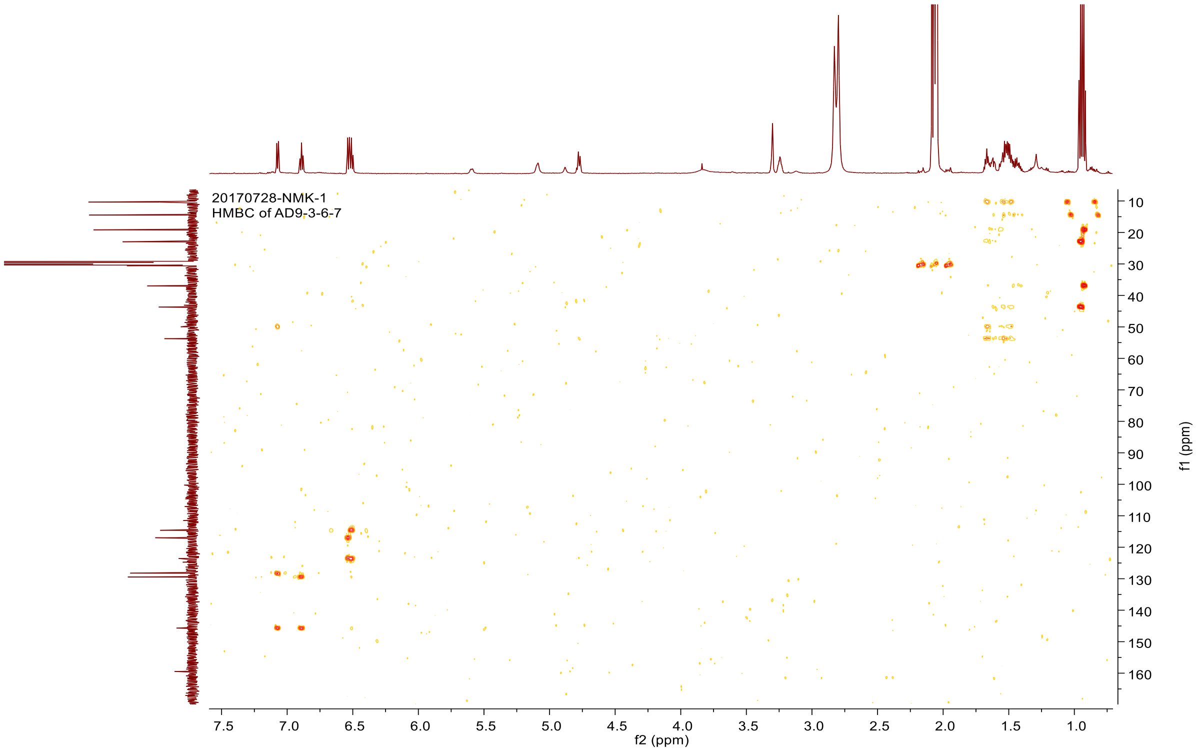

Figure S28. HMBC spectrum of compound 3 in acetone-d_6 (600 MHz)

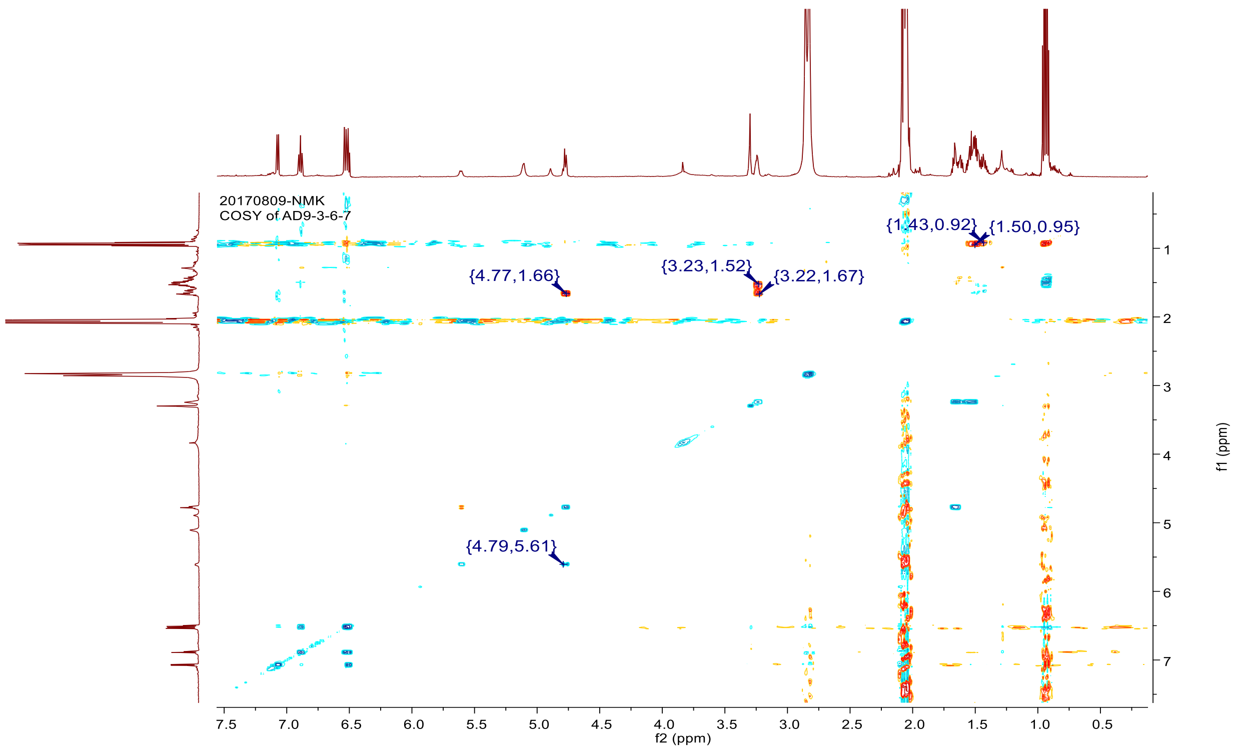

Figure S29. COSY spectrum of compound 3 in acetone-d_6 (600 MHz)

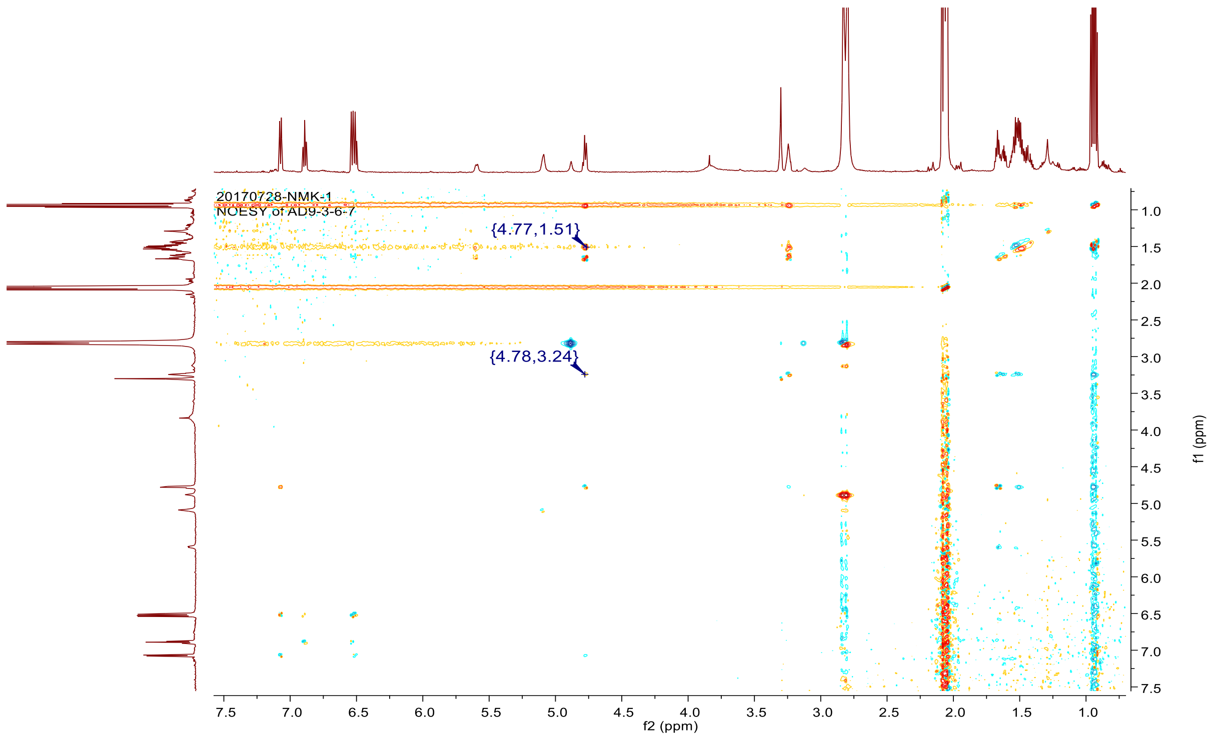

Figure S30. NOESY spectrum of compound 3 in acetone-d_6 (600 MHz)

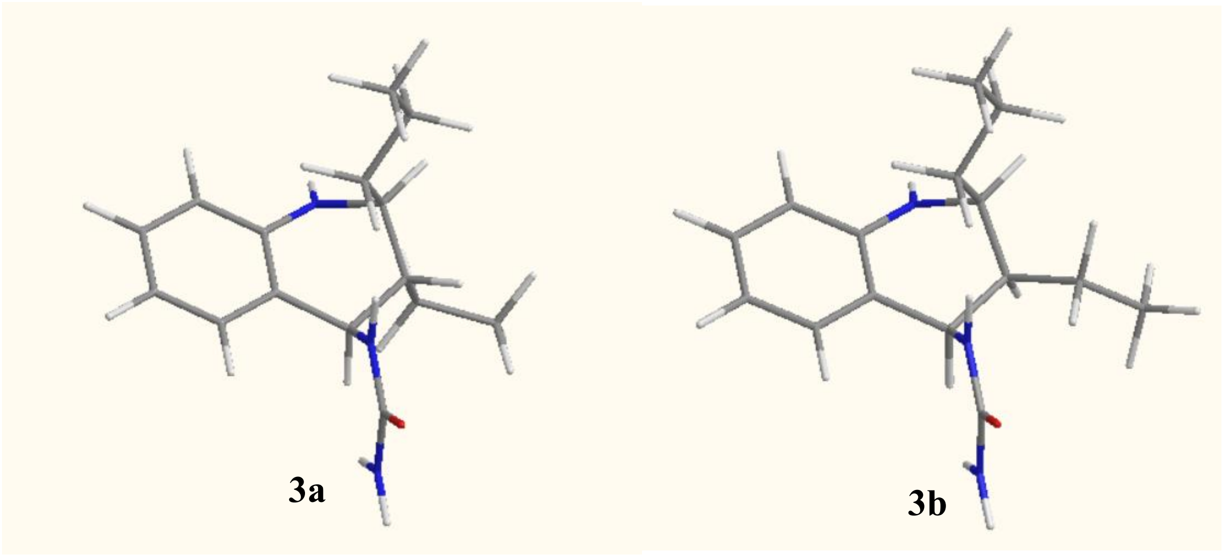

Figure S31. Candidate diastereomers 3a and 3b

Table S1. The major conformers of diastereomers of compound 3

Conformers	Boltzmann population (%)	Relative Energy (KJ/mol)
Diastereomer 3a_1	23.095	0
Diastereomer 3a_2	21.162	0.217
Diastereomer 3a_3	8.275	2.544
Diastereomer 3a_4	8.054	2.611
Diastereomer 3a_5	7.389	2.825
Diastereomer 3a_6	5.526	3.545
Diastereomer 3a_7	4.205	4.223
Diastereomer 3a_8	3.871	4.428
Diastereomer 3a_9	3.776	4.489
Diastereomer 3a_10	2.883	5.158
Diastereomer 3a_11	2.646	5.371
Diastereomer 3a_12	2.625	5.390
Diastereomer 3a_13	1.888	6.208
Diastereomer 3a_14	1.337	7.062
Diastereomer 3a_15	0.961	7.882
Diastereomer 3a_16	0.890	8.070
Diastereomer 3a_17	0.555	9.240
Diastereomer 3a_18	0.444	9.796
Diastereomer 3a_19	0.418	9.946
Diastereomer 3b_1_	25.472	0
Diastereomer 3b_2	15.351	1.255
Diastereomer 3b_3	8.837	2.624
Diastereomer 3b_4	8.808	2.632
Diastereomer 3b_5	5.754	3.688
Diastereomer 3b_6	5.541	3.781
Diastereomer 3b_7	3.985	4.598
Diastereomer 3b_8	3.735	4.759
Diastereomer 3b_9	2.977	5.321
Diastereomer 3b_10	2.975	5.323
Diastereomer 3b_11	2.933	5.358
Diastereomer 3b_12	2.743	5.524
Diastereomer 3b_13	2.060	6.234
Diastereomer 3b_14	2.059	6.235
Diastereomer 3b_15	1.511	7.002
Diastereomer 3b_16	1.386	7.215
Diastereomer 3b_17	0.990	8.05
Diastereomer 3b_18	0.691	8.941
Diastereomer 3b_19	0.585	9.354
Diastereomer 3b_20	0.561	9.457
Diastereomer 3b_21	0.526	9.619
Diastereomer 3b_22	0.519	9.653

Table S2. Experimental and calculated ^{1}H chemical shift value of 3 and its possible diastereomers 3a and 3b, repectively

Proton	Exp.	Cal.	
	3	3a	3b
2	3.21	3.48	3.76
3	1.67	2.05	2.18
4	4.70	4.78	5.36
5	7.04	7.66	7.84
6	6.56	7.14	7.14
7	6.93	7.60	7.57
8	6.56	7.03	7.01
9a	1.62	1.92	1.75
9b	1.56	1.64	1.63
10a	1.52	1.65	1.70
10b	1.52	1.56	1.52
11a	0.97	1.23	1.12
11b	0.97	0.98	1.30
11c	0.97	1.12	1.12
12a	1.50	1.59	1.82
12b	1.43	1.61	1.61
13a	0.97	1.13	1.40
13b	0.97	1.31	0.99
13c	0.97	1.25	1.04

Table S3. Experimental and calculated ^{13}C chemical shift values of 3 and its possible diastereomers 3a and 3b, respectively

Carbon	Exp.	Cal.	
	3	3a	3b
2	54.22	50.81	52.19
3	44.31	40.78	36.87
4	50.80	49.23	50.40
4a	123.25	114.64	117.37
5	129.54	124.06	120.22
6	117.81	109.04	109.24
7	128.83	121.66	120.80
8	115.37	107.40	106.89
8a	145.94	137.78	139.61
9	37.41	33.84	32.59
10	23.44	18.46	18.42
11	14.56	10.93	11.00
12	19.65	23.46	16.38
13	10.48	8.26	9.51
15	162.20	150.98	151.47

Functional	Solvent?	Basis Set	Type of Data
mPW1PW91	PCM	6-31+G(d,p)	Unscaled Shifts

		DP4+	100.00%	0.00%	-	-	-
**Nuclei**	**sp2?**	**Experimental**	**Isomer 1**	**Isomer 2**	**Isomer 3**	**Isomer 4**	**Isomer 5**
C	x	115.37	107.4	106.89			
C	x	128.83	121.66	120.8			
C	x	117.81	109.04	109.24			
C	x	129.54	124.06	120.22			
H	x	6.93	7.6	7.57			
H	x	6.56	7.14	7.14			
H	x	7.04	7.66	7.84			
C	x	123.25	114.64	117.37			
C		50.80	49.23	50.4			
C		44.31	40.78	36.87			
C		54.22	50.81	52.19			
C	x	145.94	137.78	139.61			
H		4.70	4.78	5.36			
H		1.67	2.05	2.18			
H		3.21	3.48	3.76			
C		37.41	33.84	32.59			
C		23.44	18.46	18.42			
C		14.56	10.93	11			
C		19.65	23.46	16.38			
C		10.48	8.26	9.51			
C	x	162.20	150.98	151.47			
H	x	6.56	7.03	7.01			
H		1.56	1.64	1.63			
H		1.62	1.92	1.75			
H		1.52	1.65	1.7			
H		1.52	1.56	1.52			
H		0.97	1.23	1.12			
H		0.97	0.98	1.3			
H		0.97	1.12	1.12			
H		1.43	1.61	1.61			
H		1.50	1.59	1.82			
H		0.97	1.13	1.4			
H		0.97	1.31	0.99			
H		0.97	1.25	1.04			

Figure S32. Results of DP4+ analysis of compound 3

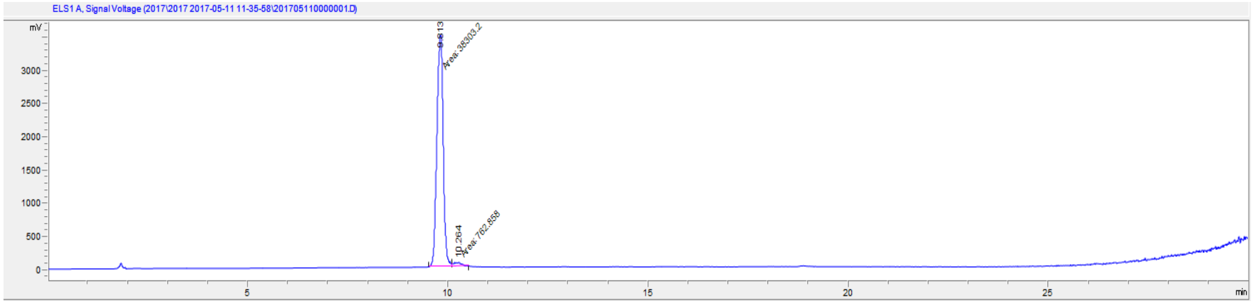

#	Time	Area	Height	Width	Area%	Symmetry
1	9.813	38303.2	3495	0.1827	98.047	1.469
2	10.264	762.9	58.4	0.2176	1.953	1.478

Purity (%) > 98%

Figure S33. Purity evaluation of 4 based on HPLC-ELSD application

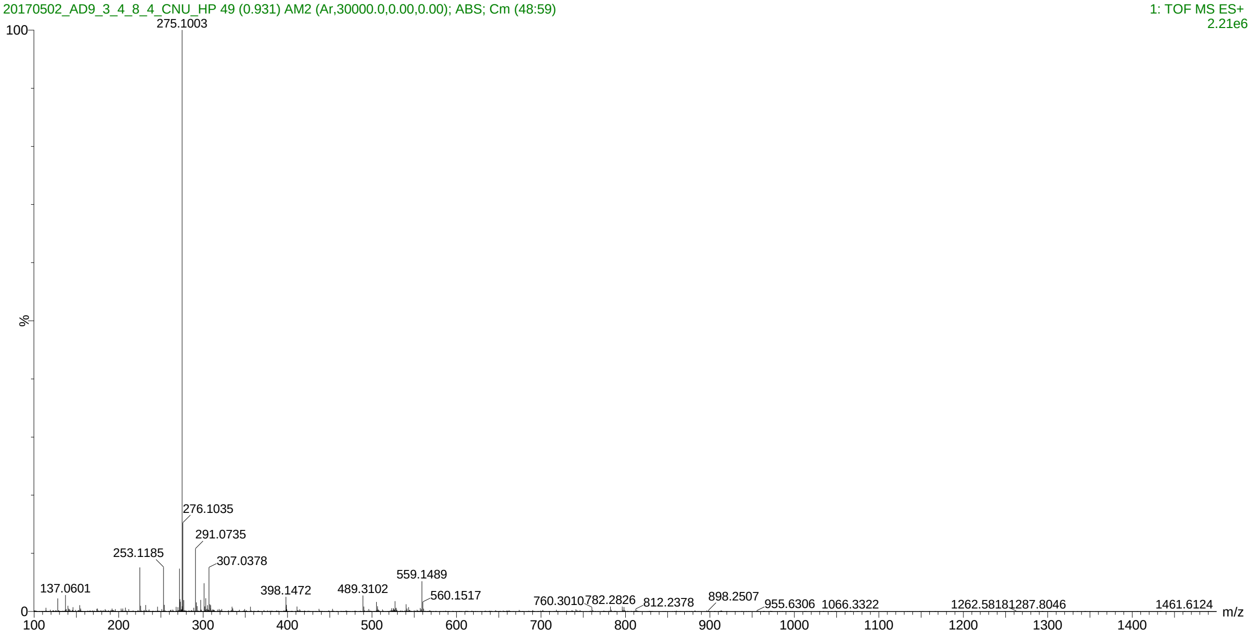

Elemental Composition Report

Mass	Calc. Mass	mDa	PPM	DBE	i-FIT		Norm	Conf(%) Formula
253.1185	253.1188	-0.3	-1.2	5.5	870.4	n/a	n/a	C12 H17 N2 O4
275.1003	275.1008	-0.5	-1.8	5.5	1261.2	n/a	n/a	C12 H16 N2 O4 Na

Figure S34. HRESIMS data of compound 4

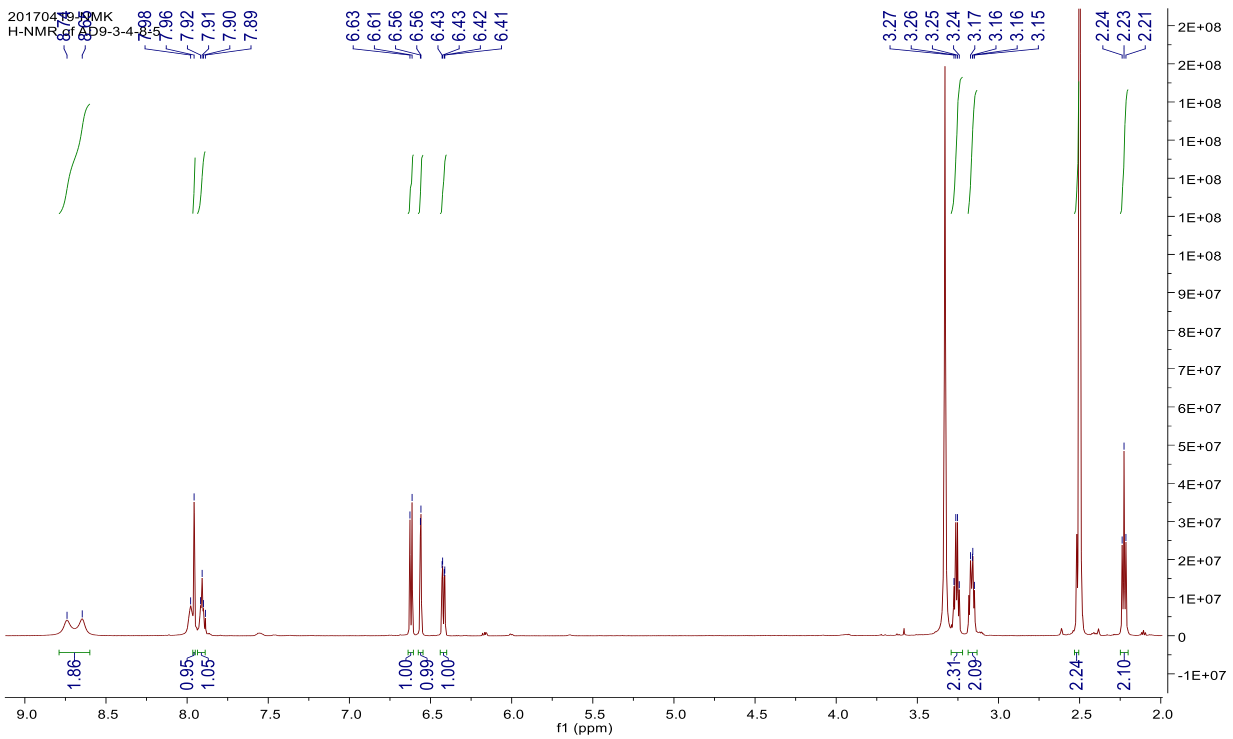

Figure S35. ^{1}H NMR spectrum of compound 4 in DMSO-d_6 (600 MHz)

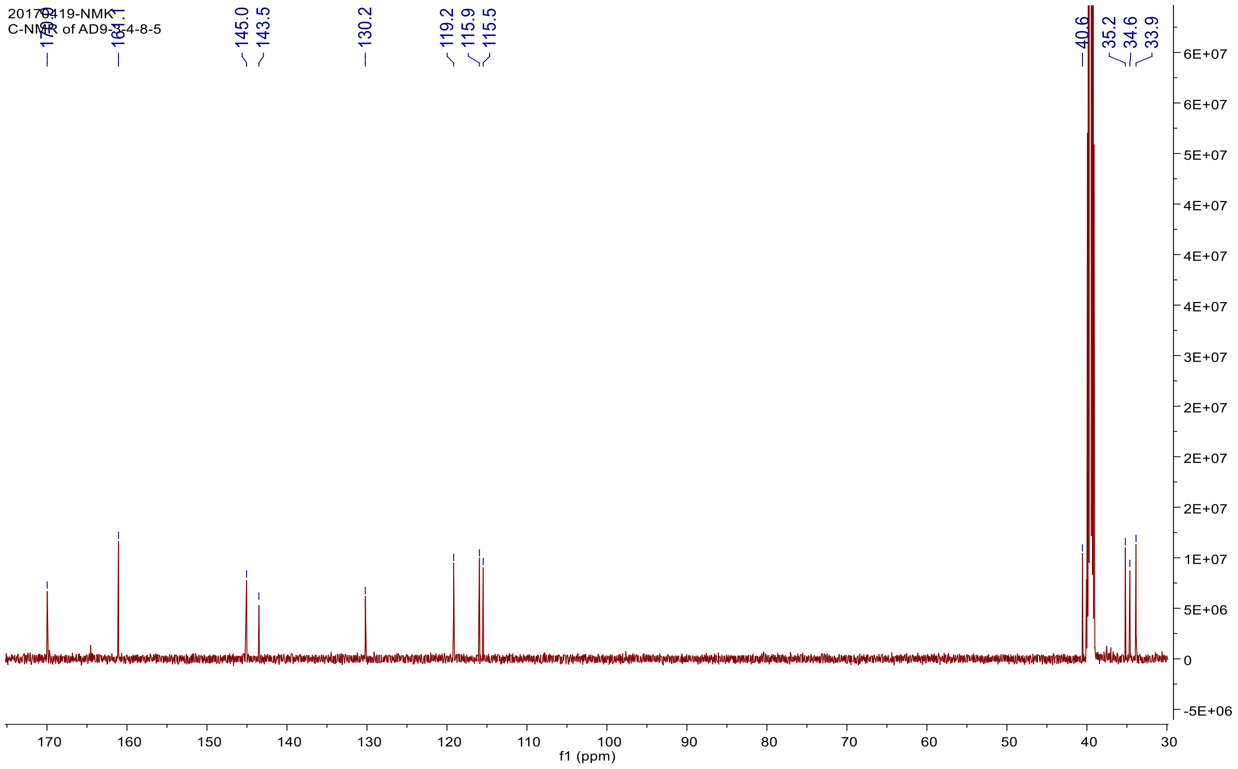

Figure S36. ^{13}C NMR spectrum of compound 4 in DMSO-d_6 (600 MHz)

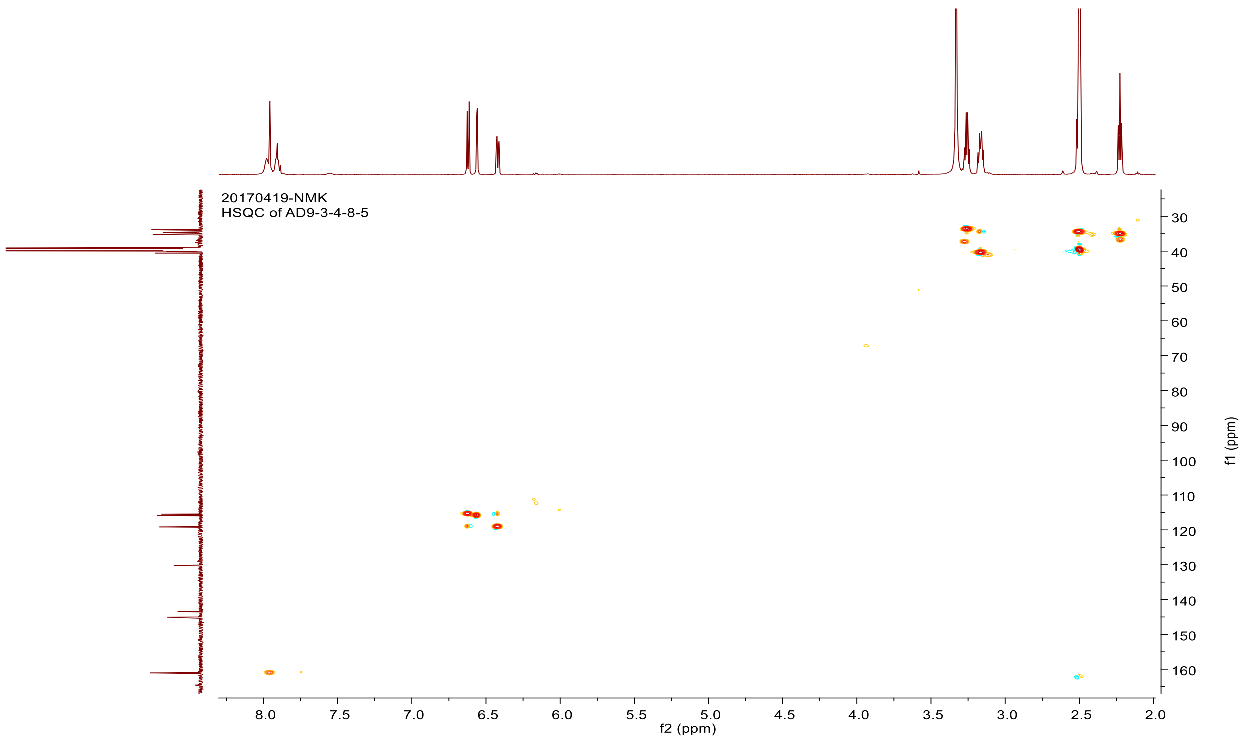

Figure S37. HSQC spectrum of compound 4 in DMSO-*d*$_6$ (600 MHz)

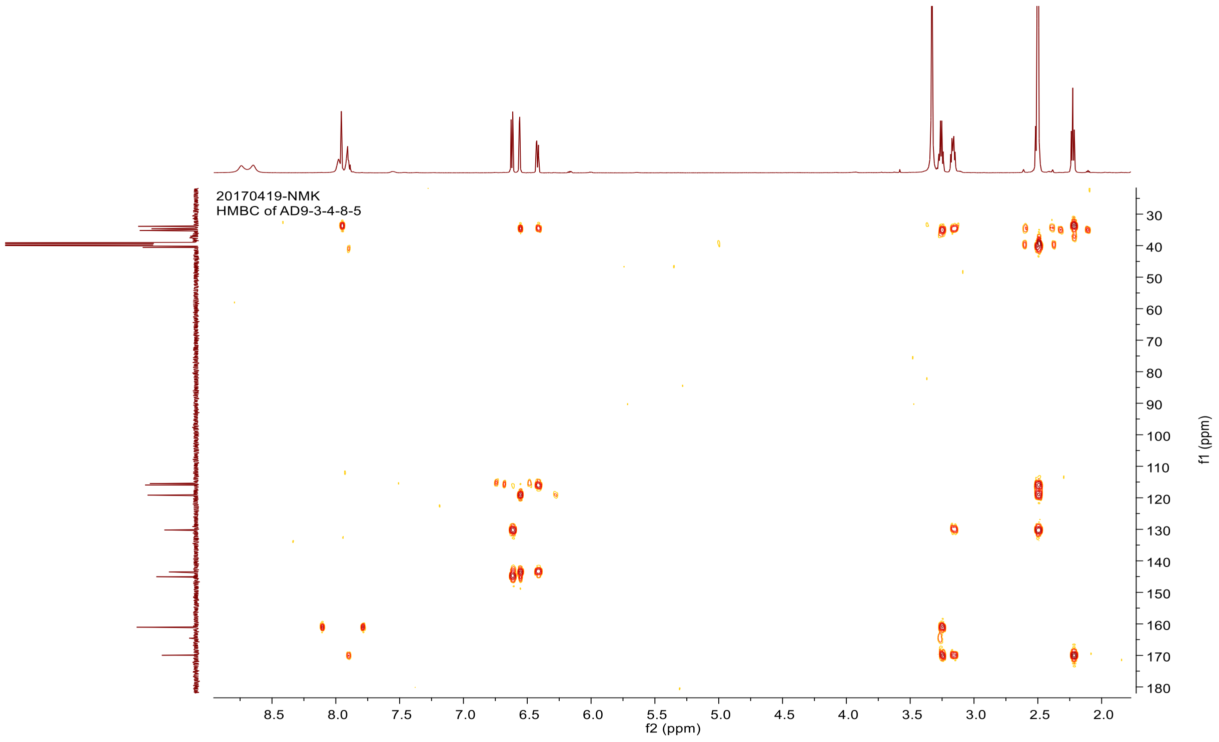

Figure S38. HMBC spectrum of compound 4 in DMSO-*d*$_6$ (600 MHz)

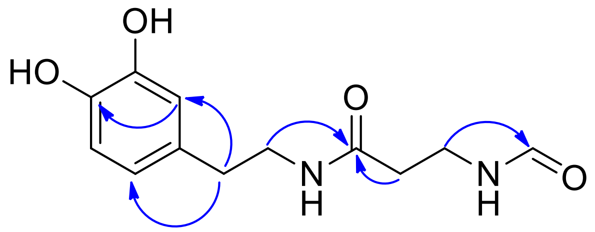

Figure S39. Key HMBC correlations of compound 4

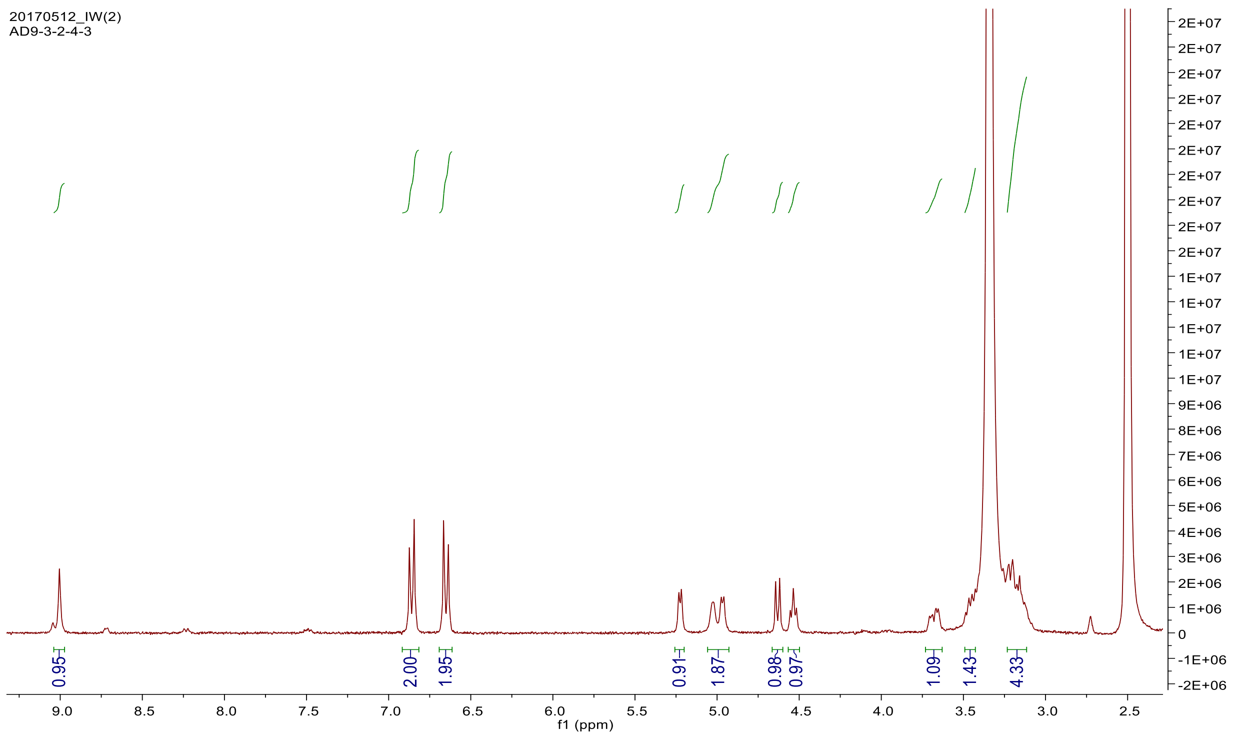

Figure S40. ^{1}H NMR spectrum of compound 5 in DMSO-d_6 (300 MHz)

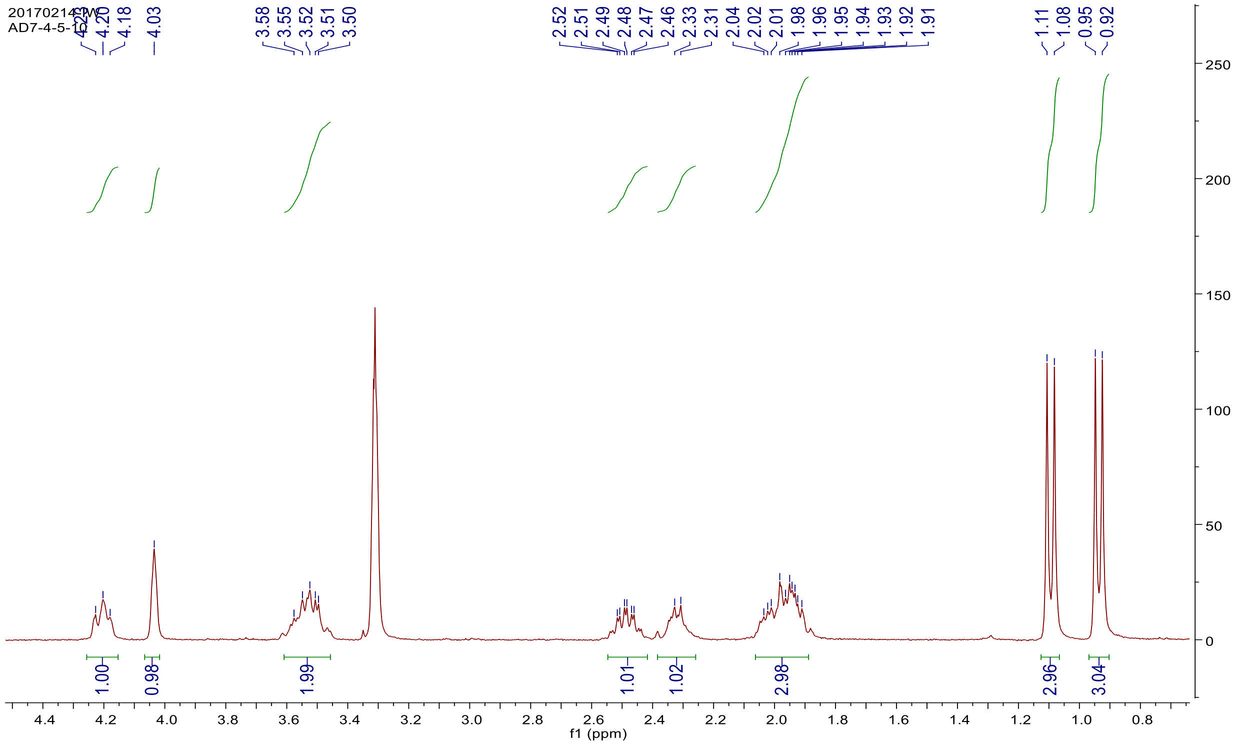

Figure S41. ^{1}H NMR spectrum of compound 6 in methanol-d_4 (300 MHz)

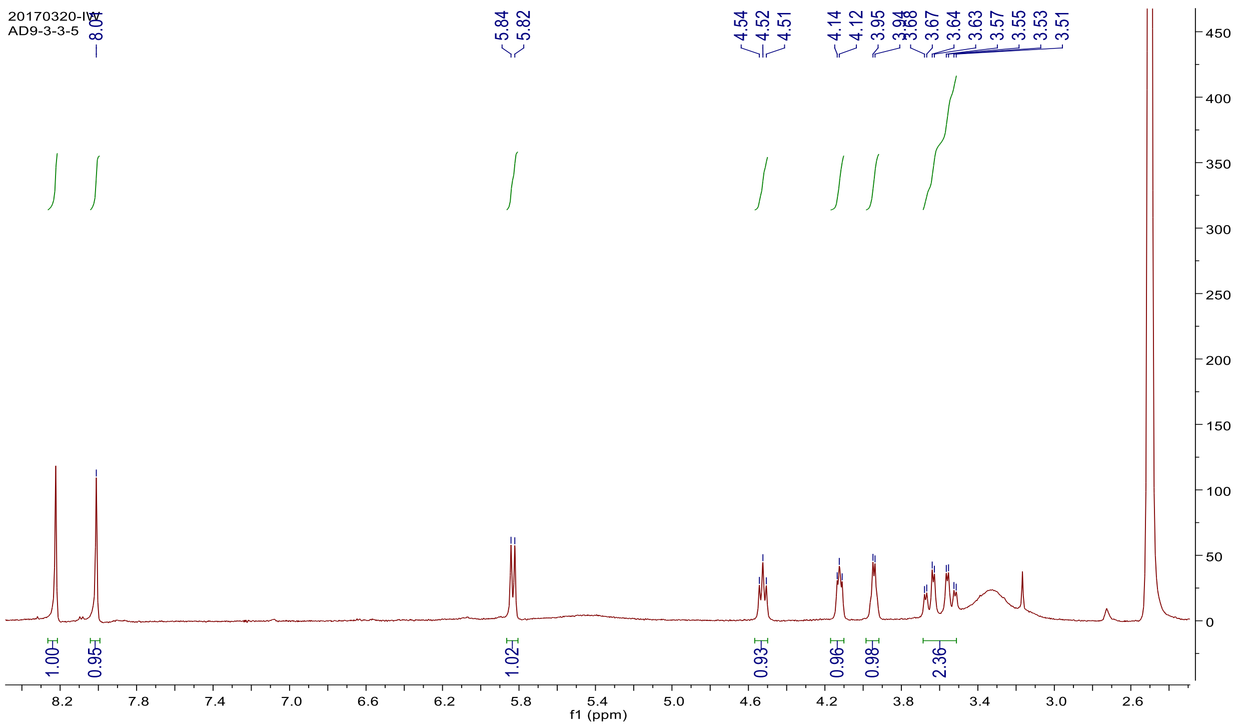

Figure S42. ^{1}H NMR spectrum of compound 7 in DMSO-d_6 (300 MHz)

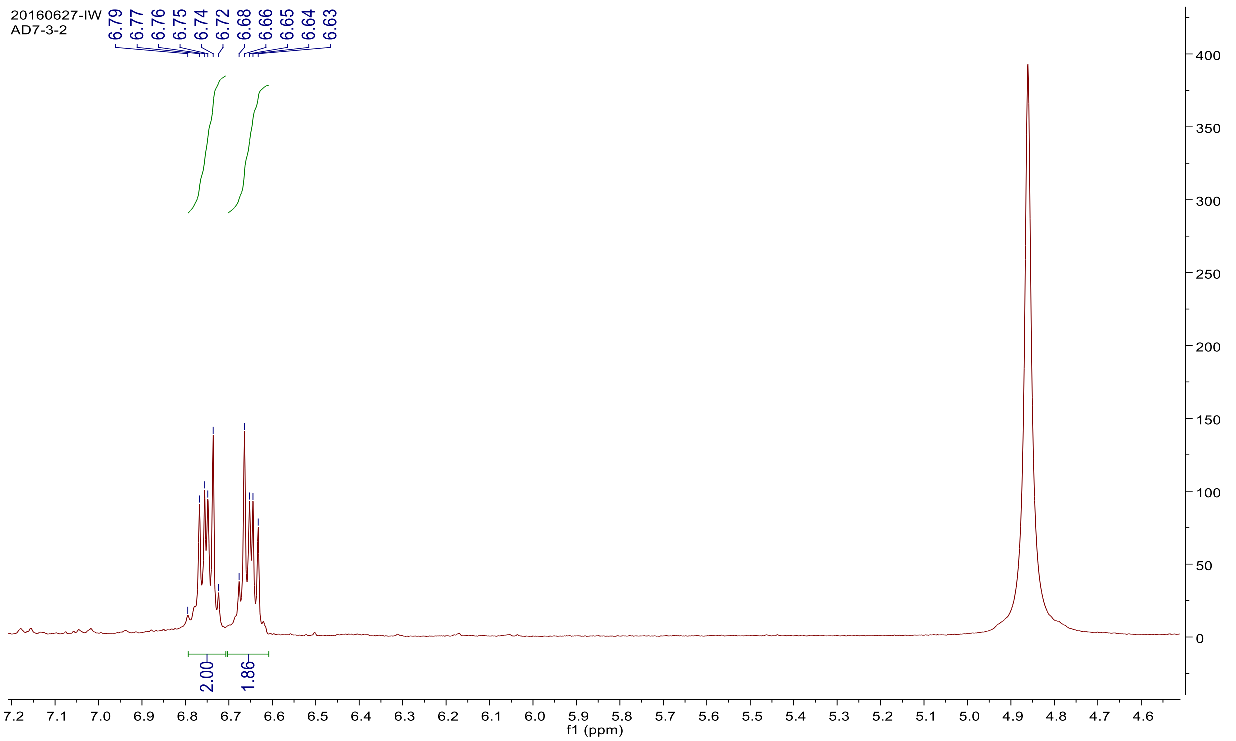

Figure S43. ^{1}H NMR spectrum of compound 8 in methanol-d_4 (300 MHz)

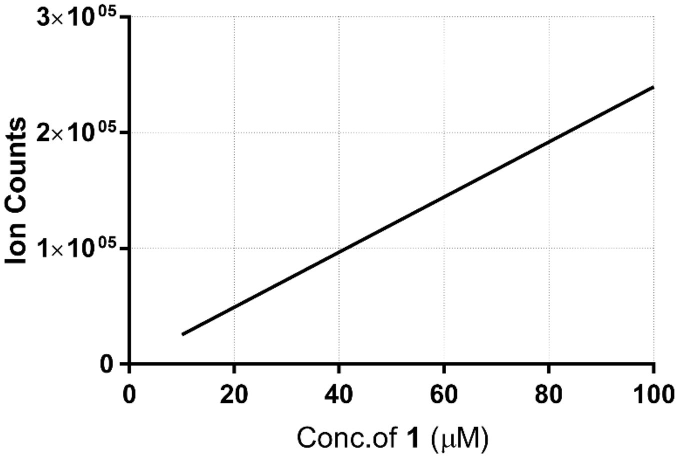

Y = 2380*X + 1640
crude: 30286.03
12 μM

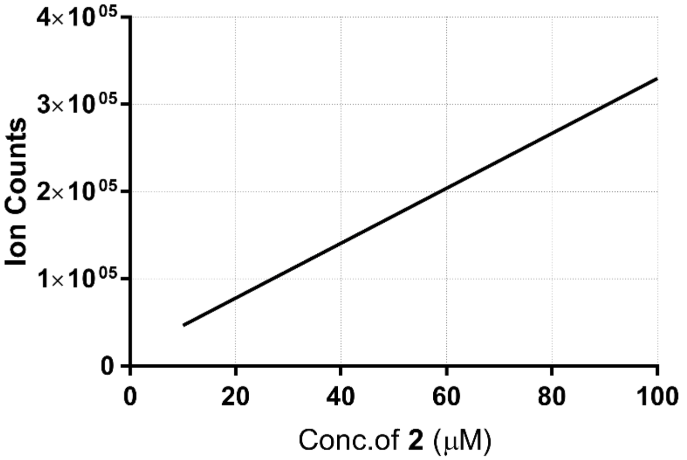

Y = 3150*X + 15026
crude: 63523.01
15 μM

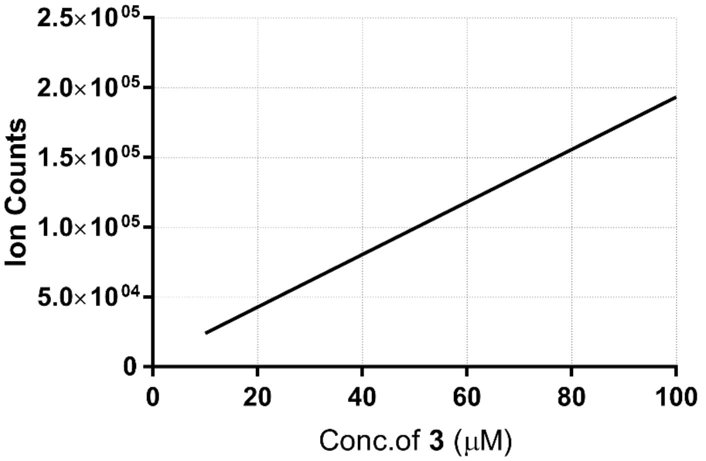

Y = 1882*X + 5113
crude: 35236.09
16 μM

Figure S44. Calibration curve of compounds 1-3 in crude extract

■ MATERIALS AND METHODS OF BIOLOGICAL ACTIVITIES

Regents. Bacterial LPS (serotype: 0111:B4, L5293), Evans blue, crystal violet, 2-mercaptoethanol, polyethylene-glycolated (PEG)-catalase, and antibiotics (penicillin G and streptomycin) were purchased from Sigma (St. Louis, MO). Fetal bovine serum (FBS) and Vybrant DiD were purchased from Invitrogen (Carlsbad, CA)

Animals and Husbandry. Male C57BL/6 mice (6–7 weeks old; average weight, 20 g) purchased from Orient Bio Co. (Sungnam, Republic of Korea) were used in this study after a 12-day acclimatization period. The animals were housed 5 per polycarbonate cage under controlled temperature (20–25°C) and humidity (40%–45% RH) and a 12:12–h light/dark cycle. Animals received a normal rodent pellet diet and water ad libitum during the acclimatization. All the animals were treated in accordance with the 'Guidelines for the Care and Use of Laboratory Animals' issued by Kyungpook National University (IRB No. KNU 2017-88).

Cell culture. Primary human umbilical vein endothelial cells (HUVECs) were obtained from Cambrex Bio Science (Charles City, IA, USA) and maintained as previously described.[1, 2] Briefly, the cells were cultured to confluency at 37°C and 5% CO2 in endothelial basal medium (EBM)-2 basal media supplemented with growth supplements (Cambrex Bio Science). All experiments were carried out with HUVEC at passage 3–5. Human neutrophils were freshly isolated from whole blood (15 mL) obtained by venipuncture from five healthy volunteers, and maintained as previously described.[1, 3]

Permeability Assay In vitro. For spectrophotometric quantification of endothelial cell permeabilities in response to increasing concentrations of each compound, the flux of Evans blue-bound albumin across functional cell monolayers was measured using a modified 2-compartment chamber model, as described previously.[1, 3] HUVECs were plated (5 × 104/well) in 3-µm pore

size, 12-mm diameter transwells for 3 days. Briefly, HUVECs were plated (5 × 104/well) in transwells with a pore size of 3 μm and a diameter of 12-mm for three days. The confluent monolayers were treated LPS (100 ng/mL, for 4 h) followed by incubation with each compound for 6 h. Transwell inserts were then washed with TBS (pH 7.4), followed by the addition of 0.5 mL of Evans blue (0.67 mg/mL) diluted in a growth medium containing 4% BSA. Fresh growth medium was then added to the lower chamber, and the medium in the upper chamber was replaced with Evans blue/BSA. 10 minutes later, the optical density of the sample in the lower chamber was measured at 650 nm.

Expression of VCAM-1 and ICAM-1. The expression of vascular cell adhesion molecule-1 (VCAM-1) and intercellular adhesion molecule-1 (ICAM-1) on HUVECs were determined by a whole-cell ELISA as described previously.[1, 3] Briefly, confluent monolayers of HUVECs were treated with each compound (10 μM) for 6 h after treatment of LPS (100 ng/mL) for 16 h. The medium was removed, cells were washed with PBS, and fixed by adding 50 µL of 1% paraformaldehyde for 15 min at room temperature. After washing, 100 µL of mouse anti-human monoclonal VCAM-1 (clone; 6C7.1) and ICAM-1 (clone; P2A4), were added. After 1 h (37°C, 5% CO2), the cells were washed 3 times, and then 100 µL of 1:2000 peroxidase-conjugated anti-mouse IgG antibody (Sigma) was added for 1 h. The cells were washed again 3 times and developed using o-phenylenediamine substrate (Sigma). Colorimetric analysis was performed by measuring the absorbance at 490 nm. All the measurements were performed in triplicate wells.

Cell-cell Adhesion Assay. Adherence of isolated human neutrophils to endothelial cells was evaluated by fluorescent labelling of monocytes as described.[1, 3] The neutrophils were labeled using 5 µM Vybrant DiD for 20 min at 37°C in phenol red-free RPMI containing 5% fetal bovine serum. Following 2 washings, the cells (1.5 × 106/mL, 200 µl/well) were resuspended in adhesion

medium (RPMI containing 2% fetal bovine serum and 20 mM HEPES) and added to confluent monolayers of HUVECs in 96-well plates, which were treated for 6 h with each compound after treatment of LPS (100 ng/mL for 4 h). The fluorescence of labeled cells was measured (total signal) using a fluorescence microplate reader (Tecan Austria GmbH, Austria). After incubation for 1 h at 37°C, the non-adherent cells were removed by washing 4 times with pre-warmed RPMI, and the fluorescent signals of adherent cells were measured by previously described methods. The percentage of adherent monocytes was calculated by the formula: % adherence = (adherent signal/total signal) × 100.

Migration Assay In vitro. Migration assays were performed in 6.5-mm diameter transwell plates containing 8-μm pore size filters, as previously described [1, 3]. Briefly, confluent HUVECs were treated with increasing concentrations of each compound for 6 h after treatment of LPS (100 ng/mL) for 4 h, followed by the addition of isolated human neutrophils to the upper compartment. Transwell plates were then incubated at 37°C in 5% CO2 for 2 h. Cells in the upper chamber were then aspirated, followed by the removal of non-migrating cells on top of the filter by using a cotton swab. THP-1 cells on the lower side of the filter were fixed with 8% glutaraldehyde and stained with 0.25% crystal violet in 20% methanol (w/v). Experiments were repeated twice per well in duplicate wells, and 9 randomly selected high power microscopic fields (HPF, 200×) were counted. The results are presented as Migration Indices.

In vivo Permeability and the Leukocyte Migration Assay. Mice were administrated with LPS (0.3 mg/mouse or 15 mg/kg, intravenously). After 4 h, the mice were intravenously treated with each compound (2.6, 5.2 μg/mouse or 0.13, 0.26 mg/kg) intravenously and injected with 1% Evans blue dye solution in normal saline. 6 h later, the mice were sacrificed and peritoneal exudates were collected by washing cavities with 5 mL of normal saline and by centrifuging at 200 g for 10 min.

The absorbance of the supernatant was read at 650 nm. Vascular permeabilities are expressed as µg of dye/mouse that leaked into the peritoneal cavity, and were determined using a standard curve, as previously described.[1, 3]

For the assessment of leukocyte migration, after the mice were sacrificed, peritoneal cavities were washed with 5 mL of normal saline. Obtained samples (20 µl) of the peritoneal fluid were mixed with 0.38 mL of Turk's solution (0.01% crystal violet in 3% acetic acid), and number of leukocytes were counted using a light microscope.

Cell Viability Assay. Cell counting kit 8 (CCK-8) from Dojindo Molecular Technologies, Inc, Kumamoto, Japan was used to measure the cell viability. Cells were grown in 96-well plates at a density of 5 × 103 cells/well. After 24 h, the cells were washed with fresh medium and treated with each compound. After a 48-h incubation period, the cells were washed, and 10 µL of CCK-8 solution and 100 µL DMEM was added, followed by incubation for 2 h. The optical density was detected at a wavelength of 450 nm by microplate reader (Tecan Austria GmbH, Austria). Cell viability was counted following manufacturer's protocol. The cell viability of the control group was assumed to be 100 %.

ELISA for NF-κB, TNF-α, IL-1β, and phospho p-38. Activity of total and Phospho-NF-κB p65 (# 7174, # 7173, Cell Signaling Technology, Danvers, MA, USA) in nuclear lysates and the concentrations of TNF-α or IL-1β in cell culture supernatants were determined using ELISA kits (R&D Systems, Minneapolis, MN, USA). Measurement of phospho p-38 expression was performed according to the manufacturer's instructions using a commercially available ELISA kit (Cell Signaling Technology, Danvers, MA, USA). The values were measured using an ELISA plate reader (Tecan, Austria GmbH, Austria).

Cecal Ligation and Puncture. The mice were anesthetized with 2% isoflurane (Forane, JW pharmaceutical, South Korea) in oxygen delivered via a small rodent gas anesthesia machine (RC2, Vetequip, Pleasanton, CA), first in a breathing chamber, then via a facemask. They were allowed to breathe spontaneously during the procedure. The CLP-induced sepsis model was prepared as described in previous studies.[4, 5] In brief, a 2-cm midline incision was made to expose the cecum and adjoining intestine. The cecum was then tightly ligated with a 3.0-silk suture at 5.0 mm from the cecal tip and punctured once using a 22-gauge needle for induction of high grade sepsis.[6] It was then gently squeezed to extrude a small amount of feces from the perforation site and returned to the peritoneal cavity. The laparotomy site was then sutured with 4.0-silk. In the sham control mice, the cecum was exposed, but not ligated or punctured, and returned to the abdominal cavity. This protocol was approved by the Animal Care Committee at Kyungpook National University prior to conduct of the study (IRB No. KNU 2017-88).

Hematoxylin and Eosin Staining. Male C57BL/6 mice underwent CLP and were administered each compound (0.13 or 0.26 mg/kg) intravenously 12 h and 50 h after CLP (n = 10). They were sacrificed 96 h after CLP. To analyze the changes in the lung morphology, lung samples were removed from each mouse, washed three times with PBS (pH 7.4) to remove any remaining blood, and fixed in 4% formaldehyde (Junsei, Tokyo, Japan) in PBS (pH 7.4) for 20 h at 4 °C. After fixation, the samples were dehydrated using serial dilutions of ethanol, embedded in paraffin, sectioned at 4 μm, and placed on a slide. The slides were deparaffinized in an oven at 60 °C, rehydrated, and stained with hematoxylin (Sigma-Aldrich). To remove the excess stain, the slides were quickly dipped three times in 0.3% acid alcohol, and counterstained with eosin (Sigma-Aldrich). The excess stain was removed using serial dilutions of ethanol and xylene, and then the slides were cover-slipped. Examination of the lung specimens was conducted by a blinded

observer under a light microscope to evaluate the pulmonary architecture, tissue edema, and infiltration of inflammatory cells, as previously defined.[7]

Measurement of Organ Injury Markers. The levels of AST, ALT, BUN, creatinine, and LDH in fresh serum isolated from the plasma of septic mice were measured using biochemical kits (Mybiosource, San Diego, CA, USA). The absorbance values were measured using a microplate reader.

Statistical Analysis. All experiments were performed at least five independent times. Student's t-test was used for comparisons, and the values were expressed as the means ± standard deviation (SD). Kaplan–Meier survival analysis was performed to determine the overall survival rates. SPSS for Windows, version 16.0 (SPSS; Chicago, IL, USA) was used to perform all statistical analyses. Statistical significance was accepted at p values < 0.05.

■ REFERENCES

1. Kim, J. E.; Lee, W.; Yang, S.; Cho, S. H.; Baek, M. C.; Song, G. Y.; Bae, J. S., Suppressive effects of rare ginsenosides, Rk1 and Rg5, on HMGB1-mediated septic responses. *Food Chem. Toxicol.* **2019,** 124, 45-53.
2. Lee, I. C.; Bae, J. S., Pelargonidin protects against renal injury in a mouse model of sepsis. *J. Med. Food* **2019,** 22, 57-61.
3. Lee, W.; Park, S. Y.; Yoo, Y.; Kim, S. Y.; Kim, J. E.; Kim, S. W.; Seo, Y. K.; Park, E. K.; Kim, I. S.; Bae, J. S., Macrophagic stabilin-1 restored disruption of vascular integrity caused by sepsis. *Thromb. Haemost.* **2018,** 118, 1776-1789.
4. Bae, J. S.; Lee, W.; Nam, J. O.; Kim, J. E.; Kim, S. W.; Kim, I. S., Transforming growth factor beta-induced protein promotes severe vascular inflammatory responses. *Am. J. Respir. Crit. Care Med.* **2014,** 189, 779-786.
5. Wang, H.; Liao, H.; Ochani, M.; Justiniani, M.; Lin, X.; Yang, L.; Al-Abed, Y.; Metz, C.; Miller, E. J.; Tracey, K. J.; Ulloa, L., Cholinergic agonists inhibit HMGB1 release and improve survival in experimental sepsis. *Nat. Med.* **2004,** 10, 1216-1221.
6. Rittirsch, D.; Huber-Lang, M. S.; Flierl, M. A.; Ward, P. A., Immunodesign of experimental sepsis by cecal ligation and puncture. *Nat. Protoc.* **2009,** 4, 31-36.
7. Ozdulger, A.; Cinel, I.; Koksel, O.; Cinel, L.; Avlan, D.; Unlu, A.; Okcu, H.; Dikmengil, M.; Oral, U., The protective effect of *N*-acetylcysteine on apoptotic lung injury in cecal ligation and puncture-induced sepsis model. *Shock* **2003,** 19, 366-372.
